# Multiple imputation approaches for epoch-level accelerometer data in trials 

**DOI:** 10.1177/09622802231188518

**Published:** 2023-07-31

**Authors:** Mia S Tackney, Elizabeth Williamson, Derek G Cook, Elizabeth Limb, Tess Harris, James Carpenter

**Affiliations:** 1 Department of Medical Statistics, 4906 London School of Hygiene and Tropical Medicine, UK; 2 Population Health Research Institute, St George’s, University of London, UK; 3 MRC Clinical Trials Unit at University College London, UK

**Keywords:** Missing data, multiple imputation, accelerometer, physical activity trial, wearables

## Abstract

Clinical trials that investigate physical activity interventions often use accelerometers to measure step count at a very granular level, for example in 5-second epochs. Participants typically wear the accelerometer for a week-long period at baseline, and for one or more week-long follow-up periods after the intervention. The data is aggregated to provide daily or weekly step counts for the primary analysis. Missing data are common as participants may not wear the device as per protocol. Approaches to handling missing data in the literature have defined missingness on the day level using a threshold on daily weartime, which leads to loss of information on the time of day when data are missing. We propose an approach to identifying and classifying missingness at the finer epoch-level and present two approaches to handling missingness using multiple imputation. Firstly, we present a parametric approach which accounts for the number of missing epochs per day. Secondly, we describe a non-parametric approach where missing periods during the day are replaced by donor data from the same person where possible, or data from a different person who is matched on demographic and physical activity-related variables. Our simulation studies show that the non-parametric approach leads to estimates of the effect of treatment that are least biased while maintaining small standard errors. We illustrate the application of these different multiple imputation strategies to the analysis of the 2017 PACE-UP trial. The proposed framework is likely to be applicable to other digital health outcomes and to other wearable devices.

## 1. Introduction

Wearable devices are increasingly becoming popular tools to measure health outcomes in clinical trials. In trials that investigate interventions aimed to increase physical activity, accelerometers have been used in a number of studies to evaluate impact on participants’ step count.^3,2,1,4^ Accelerometers measure acceleration in three dimensions in very fine intervals of time, typically in 5-second intervals or epochs, and offer a more objective measure of physical activity with reduced participant burden compared to self-report approaches. Outputs of interest from accelerometers include vector magnitude (VM), which summarizes the accelerations in three dimensions, step count and time spent in different physical activity intensities (e.g. sedentary, light, moderate-to-vigorous physical activity).^5^ Missing data can occur in a number of ways in this setting; for example, there may be device failure due to the battery running out or water damage, or participants may remove or forget to wear the accelerometer for periods of time during the day. Analyses should account for the missingness in a suitable way in order for results to be unbiased and to reflect the uncertainty appropriately. Multiple imputation (MI) is a flexible and powerful approach to handling missing data, and has previously been applied to the accelerometer setting where outcomes are aggregated at the day level.^6^

**Figure 1.**
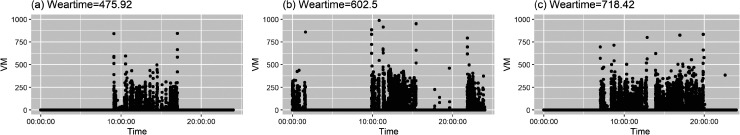
Vector magnitude (VM) is plotted against time for data from three days from three different individuals from the PACE-UP trial.

Approaches which apply MI to day-level step counts require missingness to also be determined at the day-level. A popular approach in the literature is to define a day as missing if a participant wore the device for less than 540 minutes in a day.^1,2,3,4^ Other common choices of threshold include 360 minutes of wear time^7^ and 600 minutes of weartime.^8,9^ Defining missingness at the aggregate day-level has some drawbacks. Participants may provide valuable data on the so-called ‘missing days’ (e.g. days with less than 540 minutes of weartime) which would then become discarded; for example, the plot of Figure 1(a) displays VM from a day where the device was worn for 475.92 minutes which is slightly short of the required weartime. Equally, participants who do provide at least 540 minutes of weartime could potentially still have missing parts of days; for example, the plots of Figure 1(b) and (c) are examples of days where weartime is above the 540 minutes threshold, but there are periods during the day where no data is recorded and could potentially be missing. Tackney et al.^6^ proposed an alternative approach where days are classified as missing, partially observed or observed, and partially observed days are treated as right-censored data, which retains the information from days where participants provide some, but insufficient, data. However, even with this approach, information on times of day where missingness takes place is discarded. Examining the time of day when participants have missing data can be valuable in trying to restore information and can lead to greater clarity. How to apply MI using epoch-level data has received limited attention in the literature so far.

MI is a flexible and powerful approach to handling missing data. MI accounts for uncertainty in the missing values, and allows for sensitivity analysis to explore departures from the missing at random (MAR) assumption. Further, MI can allow for outcomes to be on an aggregate level (day- or week-level), while missingness is handled on the finer epoch-level. To date, there has been limited exploration of missingness on the epoch-level data in the literature. Lee and Gill^10^ proposed a zero-inflated Poisson and log-normal mixture distribution which allows for imputations at the epoch-level. Butera et al.^11^ proposed a non-parametric (hot deck) approach to MI at the epoch-level. When data are MAR, their simulation studies showed that the non-parametric approach produced less bias and improved coverage compared to available case and complete case analyses. However, neither of these epoch-level approaches to imputation fully reflect the complexity of defining missingness at the epoch-level. Lee and Gill^10^ assumed a window of between 9 a.m. and 9 p.m. in which participants are awake, and define missingness as intervals of at least 20 minutes of no recorded acceleration. Limiting the window of data considered between 9 a.m. and 9 p.m. ignores the variation within and between people’s waking and sleeping times, and does not acknowledge the possibility that some of these periods may actually be due to the participant removing their device during sleep, which is per-protocol and should not be imputed. In their simulation studies, Butera et al.^11^ induced missingness in 2-hour blocks of time, which simplifies the complexity of missingness in genuine datasets.

This study aims to characterize the common patterns of missing accelerometer data at the epoch-level, and handle epoch-level missingness utilising MI using both parametric and non-parametric approaches, illustrated by a specific trial example. We first describe the PACE-UP trial in Section 2, and then describe Multiple Imputation and the challenges in its application in the accelerometer context in Section 3. We then describe the non-parametric and parametric approaches in Section 4. These approaches are validated through simulation studies in Section 5, and their performances are compared in an application to the PACE-UP trial in Section 6.

## 2. PACE-UP trial

We illustrate the complexities due to missing data in the accelerometer context using the 2017 PACE-UP trial as a motivating example. The PACE-UP trial investigated postal and nurse-supported interventions for increasing physical activity in patients aged 45–75 years from seven primary care practices in London. Randomization was by household (to avoid couple contamination) and block randomization was used within seven primary care practices. Of the 1023 patients in the trial, 338 were randomized to usual care, 339 to postal intervention and 346 to nurse-supported intervention. The participants were provided with an ActiGraph GT3X accelerometer (ActiGraph, FL, USA) for a period of seven consecutive days on four separate occasions, which we refer to as *measurement periods*: baseline, 3 months, 12 months, and 3 years. They were instructed to wear the accelerometer on the hip using a belt during waking hours, except when swimming or showering. The protocol and results of the trial have been reported previously,^2,1^ and the trial showed that physical activity increased in both intervention groups compared to usual care.

In the reported trial results, days are defined as missing if weartime is less than 540 minutes.^2^ Further, at least 5 non-missing days at baseline and at least one non-missing day at 12 months are required to be included in the primary analysis. Of the 1023 patients who were randomized, 93% of participants were included in the 12-month primary analysis. The average of the non-missing days were computed at baseline and 12 months to assess change in step count, adjusting for day of week, and day-order-of-wear. The primary analysis assumes that the data are MAR, and sensitivity analyses were conducted to assess the impact of using different thresholds on weartime for defining missingness, and to assess the impact of data being missing not at random (MNAR).^2^

## 3. Multiple imputation

MI is a flexible and practical approach to the analysis of datasets with missing values. An imputation model is specified, which is a model for the posterior predictive distribution of the missing outcomes given the observed data.^12^ This model is used to impute missing data with M plausible values, resulting in a total of M sets of complete data. The analysis model is fitted to each of the M datasets, and the estimates are combined using Rubin’s rules^13^ to take full account of the uncertainty due to the missing values. If the imputation model is specified appropriately, MI provides valid and efficient inference under the assumption that the data are MAR given the observed data in the imputation model.^14^ ${}^{\mathrm{Ch}.\;2}$ Sensitivity analysis to assess the robustness of the results to missing data assumptions is recommended.^15^ An attractive feature of MI in the accelerometer setting is that the imputation model and the analysis model are separate, which allows missingness to be defined on a different level than the level specified in the analysis model. For example, the analysis model may have as the outcome the step counts averaged across the week and the imputation model may handle missingness at the finer day- or epoch-level to achieve more precise imputations.

The imputation model is typically specified as an explicit parametric model for the predictive distribution of the missing variables given the observed data. For example, a multivariate normal model can be specified, or Tobit regression may be used in the accelerometer setting to incorporate step counts as right-censored observations in the imputation model if the data from participants are only partially observed. A parametric imputation model may include additional auxiliary variables which are not in the analysis model, but are predictive of missingness or step count. The inclusion of variables such as daily weather variables in the accelerometer setting can help to make the MAR assumption more plausible.^6^

An alternative approach is to use non-parametric or hot-deck imputation, which replaces missing values with donor data, which are observed data that have been identified to be of similar characteristics to the missing data.^16^ Identifying such data involves consideration of the variables required to make the MAR assumption plausible. An important advantage of this approach is that it is compatible with complicated relationships in the dataset which do not have to be specified via a statistical model.^14^ ${}^{p.\;181}$

Prior to implementing either parametric or non-parametric MI in the accelerometer data context, however, two important challenges need to be addressed: firstly, the challenge of defining missingness at the epoch-level, and secondly, the challenge of handling difficult distributions. These are discussed in the next section.

### 3.1. Complications for MI in the accelerometer context

#### 3.1.1. Challenge 1: Defining missingness at the epoch-level

The first challenge in defining missing data in the accelerometer context is identifying when participants have removed the device. For some wearable devices, additional data from other measurements, such as heart rate, may be available to aid in identifying device removal. Heart rate would be measured when a participant is not moving while wearing the device, but missing if the participant has removed it. However, the GT3X+ accelerometers used in the PACE-UP trial does not measure heart rate. It is therefore difficult to decide whether a period with no movement recorded is (A) a period where the participant is wearing the device but staying still, (B) a period where the participant has removed the device per protocol, such as during sleep, or (C) whether the participant has removed the device during the day and is an instance of missing data. Participants’ movement is often quantified by Vector Magnitude (VM), which is the square root of the sum of the acceleration in each component squared. Figure 1 displays the plots of VM against time for 3 days from different participants in the PACE-UP trial. In plot (c), there is a short period of no activity in the middle of the day, which could be missing data, or perhaps could be the participant lying still. In contrast, the longer periods of no movement in the morning and in the evening are very likely to be the participant removing the device for sleep, as per protocol. There is a need to classify these types of activities at the epoch-level, in order to identify the missing intervals that need to be imputed through MI.

#### Challenge 2: Difficult distributions

Accelerometer data at the epoch-level are characterized by a large proportion of zeros, a heavy positive skew, and high autocorrelation.^10^ The complexity in the distribution of epoch-level data means that parametric approaches to imputation that assume a standard distribution, such as the normal distribution, are likely to be inappropriate.

Further, epoch-level accelerometer data collected over the course of a week are characterized by complex within-person patterns. There are patterns of activity that are dependent on time of day, and these patterns are generally different on weekends compared to weekdays. Allowing for these patterns on the epoch-level is a statistical and computational challenge.

## 4. Proposed approaches

### 4.1. Classifying missingness at the epoch level

Resolving the first challenge of identifying missing periods begins by identifying *zero-count* periods. Zero-count periods are intervals of time where VM is continuously zero over a specified threshold, usually set at 20, 60, or 90 minutes, where it can be assumed that the device is removed.^17^ Some authors recommend allowing for a *spike tolerance*, that is, allowing for an interval of up to 2 minutes of non-zero VM to account for inadvertent movements of the device, such as the device being moved across the table.^18^ We adopt the definition used in the PACE-UP trial, where a 60-minute threshold was used, allowing for a 2-minute spike tolerance. We note that zero-count periods are sometimes referred to as *non-wear* in the literature, but we reserve *non-wear* to refer more specifically to periods where people are likely to have removed the device.

**Figure 2.**
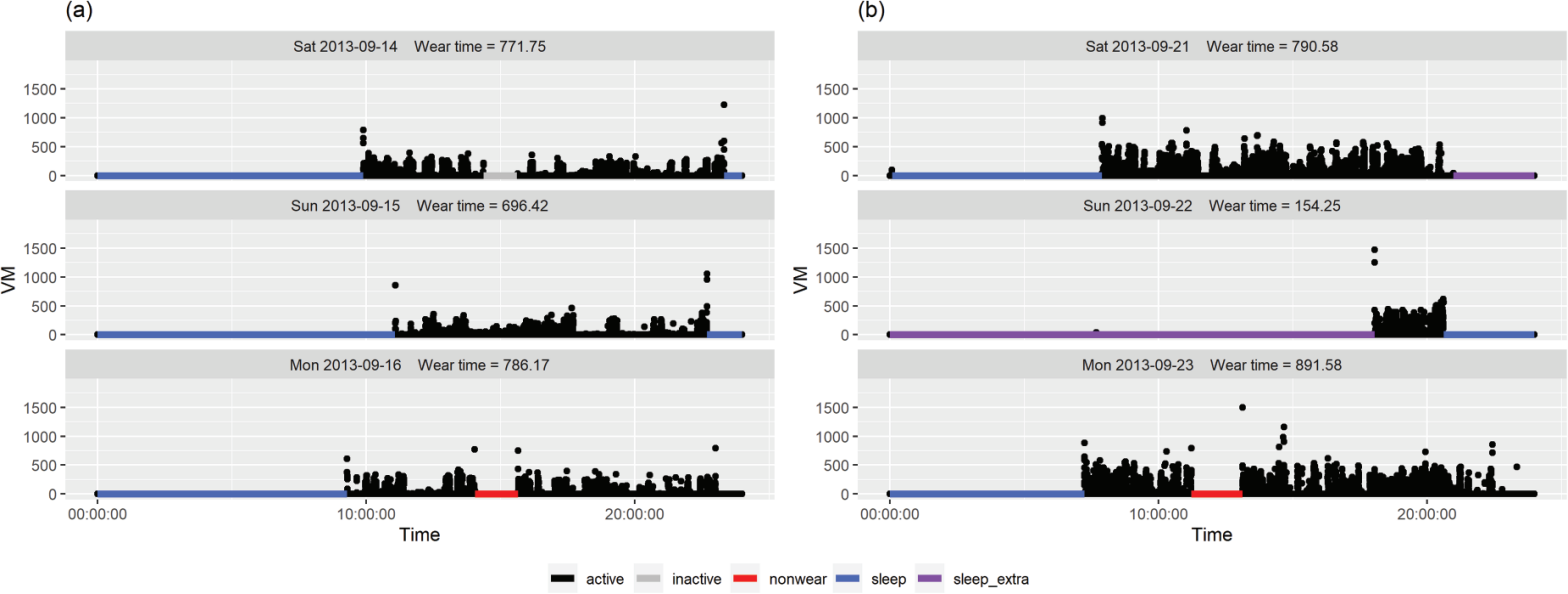
Vector magnitude is plotted against time for two individuals. In (a), we observe an *inactive* period on Saturday and a *non-wear* period on Monday. In (b), we observe 3 days, where the first and third days display sufficient weartime but on the second day, the accelerometer was not worn for the most part of the day, resulting in a *sleep-extra* period.

Zero-count periods include periods where participants are wearing the device, but are staying still. In order to distinguish these periods, we note that putting on and removing the device requires a sharp movement, which is detectable as a spike in VM. Empirically, we explored the data and confirmed that this is the case; VM greater than 600 is typically incurred when the accelerometer is put on or removed. We classify zero-count periods lasting between one and five hours with VM of at least 600 in the 2-minute interval before or after the period, as *non-wear* periods. For example, in the panel of Figure 2(a), we observe on Monday (the bottom graph) a zero-count period indicated in red where the high VM points immediately before and after indicate that the device was removed, so we classify this as non-wear. In contrast, on Saturday (the top graph), we observe a zero-count period, indicated in grey, where no high VM is detected before or after. In this case, it is possible that the participant is still wearing the device, but staying very still. We classify zero-count periods of up to 3 hours, where no high VM are detected, as *inactive* periods, which are not treated as missing.

Missingness also occurs when people put on the device later in the day than when they are expected to wake up, or remove the device earlier than when they are expected to go to bed. This is visible by a very long period zero-count period which would include the time when the participant is expected to be asleep. For example, in the panel of Figure 2(b), the purple area illustrates a case where the participant has not worn the device until late in the evening. We refer to these extended zero-count periods, lasting longer than 15 hours, as *sleep-extra* periods. Based on these observations, we classify zero-count periods into:

- *Inactive*: a shorter continuous zero-count period, lasting between 1 and 3 hours, where no high VM is detected (VM does not exceed 600 in the 2 minutes just before/after the zero-count period). This suggests that accelerometer is still worn by the individual, but they are staying very still. During a period of 1 to 3 hours, it is plausible that a person is staying still. Inactive periods are not missing periods.
- *Non-wear*: a continuous zero-count period (lasting between 1 and 5 hours), where VM exceeds 600 in the 2 minutes just before or after the period. This suggests the accelerometer has been taken on/off. Further, any zero-count period between 3 and 5 hours is classified as non-wear, since it is less plausible that a person could stay still for an extended period of time; experience with using the accelerometer suggests that it is very unlikely that it will register no movement for over 3 hours if the device is being worn. Non-wear periods are missing periods.
- *Sleep*: A zero-count period lasting between 5 and 15 hours. Sleep periods are not missing periods.
- *Sleep-extra*: A zero-count period lasting longer than 15 hours. Such an extended period of sleep suggests that a person delayed putting on the device in the morning, and/or took it off too early in the evening. Sleep-extra periods contain missing periods.

Examples of plots displaying VM against time for the 7-day period at baseline and 12 months for specific patients are shown in the Appendix Figures 11 to 13, with zero-count periods classified.

Using this classification, days/participants who do not have non-wear or sleep-extra periods are considered fully observed. Non-wear and sleep-extra periods lead to missing periods that need to be accounted for in the analysis. For non-wear periods, the start and end times of the missing period are equal to the start and end times of the non-wear period. However, for sleep-extra periods, which include per-protocol sleep periods which are not considered missing, the start and/or end times need to be estimated. If the sleep-extra period for a participant falls on a weekday, the *average sleep window* for weekdays is computed by taking weekdays from this participant with completely observed data, and finding the interval between the average time at which the participant goes to sleep, and the average time at which the participant wakes up. The missing intervals consist of the sleep-extra period, minus the period which lies in the average sleep window. If the missing interval falls on a weekend, then, if the other weekend day is fully observed, the average sleep window of that weekend day is used to compute the missing intervals. If both weekend days have missingness, the average sleep window is obtained from the weekdays, adding an empirically-based estimate of the shift in waking times at the weekend compared to weekdays. Since this was approximately an hour in these data, we rounded it to exactly one hour for convenience.

We note that this approach to classifying zero-count periods relies on assumptions about the length of time that people sleep and the length of time that people can be inactive while still wearing the accelerometer. Investigating the validity of our chosen thresholds is an important area of future work. These thresholds were proposed for our analysis of studies for physical activity trials in healthy populations, but will need to be revised for studies investigating different populations, such as children or participants with health conditions. Ideally, a validation study in a small subset of data where intervals are classified based on participants’ self-reports is recommended, but we recognize that this is often not possible. Sensitivity analyses for the choices of threshold are therefore a more practical avenue to evaluate the impact of the thresholds.

Having defined missing periods at the epoch-level, we wish to handle missingness with Multiple Imputation. We describe two approaches to overcoming the second challenge of epoch-level data having complex distributions: a parametric approach, described in Section 4.2 and a non-parametric approach, described in Section 4.3. We introduce some notation to describe the approaches. We denote by $y_{i,j,k,l}$ the step count for patient i , at measurement period j , on day k and epoch l , and we denote by $y_{i,j,k,l_{p}:l_{q}}$ the step counts over an interval between epoch $l_{p}$ and epoch $l_{q}$ (inclusive), where $l_{p}<l_{q}$ . We assume, without loss of generality, that data are recorded in 5-second epochs. We denote by $y_{i,j,k,l_{p}:l_{q}}^{obs}$ an interval that is observed, and $y_{i,j,k,l_{p}:l_{q}}^{mis}$ an interval that is missing. We denote by $y_{i,j,k,.}$ the day-level step counts for day k , and ${\bar{y}}_{i,j,.,.}$ the mean of the daily-level step counts for measurement period j .

### 4.2. Parametric approach

In the parametric approach to MI, in order to overcome difficulty of epoch-level step count data having a high proportion of zeros and extreme positive skew, we aggregate the data to the day level. Day-level step counts still have a positive skew, but this can be handled with a log-transformation to help make the normality assumption plausible. A parametric approach to MI at the day-level was proposed by Tackney et al.^6^; we make a crucial adaptation to this approach to incorporate information about missingness at the epoch-level.

The day-level approach by Tackney et al.^6^ classified step counts as completely observed if weartime ≥540 , partially observed if 0<weartime<540 , and missing if weartime =0 . In our adapted approach, we move away from using a threshold based on weartime and take into account the missing periods detected at the epoch-level. We consider a daily step count as completely observed if there are no missing periods. A daily step count is partially observed if there are non-wear or sleep-extra periods, and completely missing if no data was recorded.

We consider the daily step counts as right-censored data if they are partially observed or missing, and use Tobit regression to conduct the imputation. Tobit regression requires specification of lower and/or upper bounds for each observation. For days that are completely observed, the lower and upper bounds are the recorded logged step counts. For days that are completely missing, the lower bound is set to zero, and the upper bound is a value higher than the highest observed logged daily step count in the data (e.g. 10.5 on the log scale). For days where some activity is observed, but which have missing periods, the lower bound is the recorded step count, and we propose a *Person-specific upper bound*, calculated as $\log\;(y_{i,j,k,.}+5\lambda_{i,j,k})$ , where $\lambda_{i,j,k}$ is the number of missing epochs for participant i at measurement period j for day k . This assumes that the upper bound of total step count would allow up to one step each second (five steps per epoch) in the missing period. This approach adjusts the upper bound according to the quantity of missingness detected at the epoch-level. We compare using the person-specific upper bound to using a *Generic upper bound*, which sets the upper bound as 10.5 on the log scale. This generic upper bound was used by Tackney et al.^6^ and serves as a comparison with previously suggested approaches.

We assume that the logged daily step counts are jointly normally distributed, possibly dependent on baseline characteristics such as sex, age and body mass index (BMI), and further, we assume that the data are MAR. Activity patterns across days are accounted for through adopting a joint model for the logged daily step counts, and through the addition of covariates. We impute separately within each trial arm. After imputation, the log of the daily step counts are exponentiated. The M complete datasets on the step count scale can then be analysed separately, with estimates combined across imputed datasets using Rubin’s rules.

We note that, if the log transformation does not sufficiently reduce skewness of daily step counts, imputation via predictive mean matching (PMM) is an alternative approach to Tobit regression which is more robust to model misspecification.^19^

### 4.3. Non-parametric approach

Secondly, we consider a non-parametric approach to using MI in epoch-level data. Instead of specifying a parametric statistical model for the distribution of the missing data given the observed data, a non-parametric approach proceeds by imputing missing periods with observed periods from the same time of day, from the same participant, but from a different day of the week, where possible.

If it is not possible to impute from the same participant due to the extent of the missingness, the interval is imputed from a different participant who is as similar as possible according to demographic variables. A number of factors need to be considered in order to identify a non-self donor. Firstly, demographic variables that determine the similarity between two participants need to be identified. These variables are typically auxiliary variables those that help to strengthen the MAR assumption. Secondly, a metric is needed to measure the strength of the similarity. It may be the case that for some variables, such as sex, it is preferable to identify a donor that matches perfectly, and for other variables, a metric such as the Mahalanobis distance is used to find the closest donor.

We assume that imputation is within a treatment arm, and within a specific time interval j . For each participant i , missing periods are identified and classified. If any missing period is spread between two days, for example between epoch $l_{p}$ on day k and epoch $l_{q}$ on day k+1 , this is split into two missing periods, $y_{i,j,k,l_{p}:17280}^{mis}$ and $y_{i,j,k+1,1:l_{q}}^{mis}$ , where 17,280 is the number of 5-second epochs in a day. We obtain the set of missing periods $I_{i}$ for participant i . We denote by $|I_{i}|$ the size of the set $I_{i}$ .

Suppose $y_{i,j,k,l_{p}:l_{q}}^{mis}$ is the g th missing period in a non-empty set of missing periods for participant i , $I_{i}$ , where $g\in \{1,\ldots ,|I_{i}|\}$ . Imputation proceeds as follows:

- obtain the self-donor pool ${SD}_{i,g}$ , which consist of observed intervals $y_{i,j,k^{\prime },l_{p}:l_{q}}^{obs}$ , where $k\neq k^{\prime }$ .
- If $|{SD}_{i,g}|>4$ , sample M times with replacement from ${SD}_{i,g}$ with equal sampling probabilities, to obtain M imputed intervals.
- If $|{SD}_{i,g}|\leq 4$ , we obtain a non-self donor from the pool of participants who do not have missing periods that overlap with the g th missing period of patient i . From this pool of participants, we match perfectly on selected discrete covariates and compute the Mahalanobis distance between patient i and other participants in the non-self donor pool for covariates selected to provide approximate matching on. Here, we match perfectly on sex and approximately on BMI and age. If imputing at 12 Months, the average step count at baseline and average weartime at baseline can additionally be included in the Mahalanobis distance. We note that imputation is conducted within a specific measurement period, so even if baseline characteristics are used to compute a Mahalanobis distance, data used in the imputation is from 12 months. We then compute sampling weights for each participant in the donor pool by taking the inverse of the Mahalanobis distance for that participant as a fraction of the sum of inverses of Mahalanobis distances from all participants in the donor pool. One donor is selected using these sampling weights. From this selected donor $i^{\prime }$ , seven observed intervals $y_{i^{\prime },j,k,l_{p}:l_{q}}^{mis}$ for k∈{1,…,7} are identified. By sampling from these seven intervals with replacement (since these donors have no missing data at that measurement period), M imputed intervals are obtained.

There may be participants with almost no data, in which case we may wish to impose a minimum threshold for performing the non-parametric imputation described above, since there would be insufficient data to compute the participant’s sleep-window and to identify the intervals that need to be imputed. If this threshold is not met, we instead impute the entire week from the pool of donors who have complete data for the whole week.

In the analysis of the PACE-UP trial, we require participants to have at least 5 days where weartime is less than 300 minutes to impute an entire week. While this does not occur at baseline (participants needed to provide at least 5 days with weartime of at least 540 minutes at baseline to be included in the study), it did occur at 12 months. Here, we discard data from this participant and impute the entire week with the pool of donors who provide complete data 12 months. Matching perfectly on sex, we obtain sampling weights, based on the inverse of the Mahalanobis distances for age, BMI, average step count at baseline and average weartime at baseline for patient i and the participants in the donor pool. We select M donors using sampling weights. For each M , we randomly select 5 weekdays with replacement and 2 weekend days with replacement to impute the missing week.

After all intervals in all non-empty intervals $I_{i}$ are imputed, M complete epoch-level datasets are formed. Analyses can be implemented on each of these complete datasets, and the results combined with mean daily step count and variance from M datasets with Rubin’s rules.

We note that the non-parametric approach requires decisions to be made about a number of factors, including: the minimum size of the self-donor pool to allow imputation via self-donation; the demographic variables to match on for non-self donation; the choice of whether to match perfectly or via a distance metric; and the threshold on weartime for full-week imputation. In our setting, we chose to impute with non-self donors if there are 4 or less intervals in the self-donor pool. In choosing 4 as our threshold, we took into account a number of factors. A higher threshold would result in non-self donation occuring more frequently, which would increase the variability of the imputed values. A lower threshold would result in many repeated imputed values, since we require 10 imputations. We recommend that these choices are investigated more formally in a series of sensitivity analyses.

In studies where sample sizes are small, the non-parametric approach may still be feasible if measurement periods are sufficiently long to allow for imputation primarily by self-donation. Where sample sizes are small and the measurement period is short, the non-parametric approach will be inappropriate, but such a setting is unlikely in clinical trials designed to study efficacy (i.e. Phase IIb or Phase III trials).

**Figure 3.**
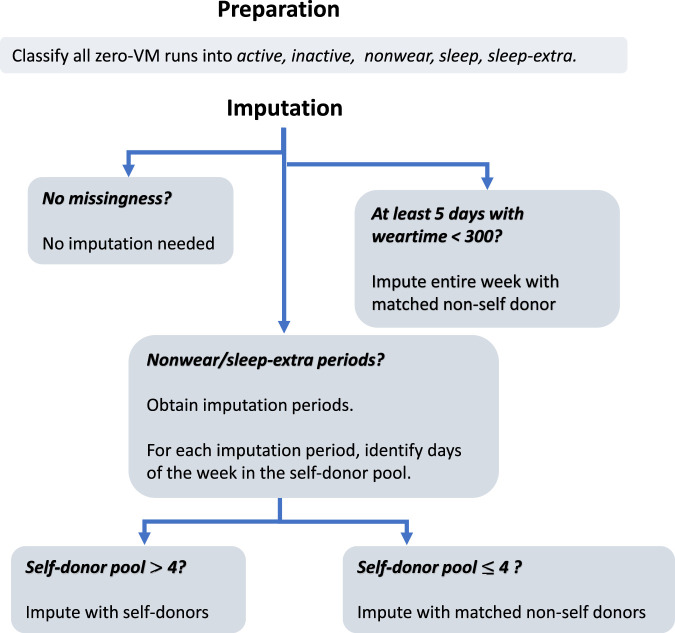
Schematic diagram for the non-parametric approach.

A simplified schematic diagram is shown Figure 3. R Code for the non-parametric approach and an associated Vignette is provided in Supplemental Materials.

## 5. Simulation studies

We conducted simulation studies to establish statistical properties of the proposed approaches to handling missing data at the epoch-level. In the first simulation, we consider the setting where there is data from one measurement period, and we wish to estimate the mean step count. In the second simulation, we consider a more complex and more common setting, where there is a treatment effect of interest, and there are two measurement periods – at baseline and after the intervention. The baseline step count is used as an adjustment variable in the treatment effect estimation.

### 5.1. Simulation 1: One measurement period

The first simulation aims to compare the statistical properties of parametric and non-parametric methods in estimating the mean and standard error of average weekly step count at one measurement period.

Step count data on the epoch-level have complex distributions that are difficult to characterize using a parametric model; devising a data generating model which adequately captures this complexity is difficult. Therefore, in our simulations, we take the approach of using bootstrap resamples from the subset of the patients in the PACE-UP trial who have complete data, inducing missingness in the data, and comparing different approaches to handling the missingness. By taking the subset of participants who have complete data as the basis of the simulation and subsequently inducing missing data patterns, we ensure that we can obtain the true value of the average weekly step count. There were 473 patients at 12 months who have completely observed data in the PACE-UP trial (164 in the control group, 162 in the postal group and 147 in the nurse group). In each repetition of the simulation study, we obtain a bootstrap sample where 120 patients in each treatment group are sampled without replacement. This creates a sample of 360 patients with complete data. Ensuring that we sample without replacement is important, since having exact copies of patients in a dataset would put the non-parametric approach at an advantage since it uses donor pools from other patients.

We then generate missingness under the following two scenarios:

- Scenario A: For a randomly selected 45% of these 360 patients, sleep-extra periods and/or non-wear periods are induced in the following way: we randomly select a patient from the PACE-UP trial who has incomplete data at 12 months, and induce sleep-extra and/or non-wear periods according to the randomly selected patient’s missingness pattern; we set the VM to zero during this time and add the high VM observed from the selected missingness pattern at the start and end of the period. This ensures that missingness is generated in a way that is representative of what is observed in a real life setting.
- Scenario B: In addition to inducing sleep-extra and/or non-wear periods in 45% of patients, we randomly delete the entire week’s step-count data for a randomly selected 2% of patients.

We then consider the following methods of handling the incomplete data:

- *Available case analysis*: As a benchmark, we analyse the data as if it were the observed data, making no attempt to handle the missingness.
- *Minimum weartime approach*: Participants who provide at least 1 day of at least 540 minutes of weartime at 12 months are included. The daily step count for any day with weartime of less than 540 minutes is set to missing. The average of the non-missing days are computed at baseline and at 12 months.
- *Non-parametric MI*, as described in Section 4.3, where age, sex and BMI are used as matching variables to sample non-self donors. We set M=10 .
- *Parametric MI*, with person-specific and generic upper bounds, as described in Section 4.2. We include BMI, sex and age as covariates in the imputation model, and set M=10 .

The estimands of interest are the mean and standard error of the average weekly step count at 12 months. We fit the following regression model for each trial arm separately (control, postal, and nurse):

(1) $${\bar{y}}_{i,1,...}=\beta_{0}+\epsilon_{i}$$

where we assume that $e_{i}∼N(0,\sigma^{2})$ , and obtain the estimate and standard error of $\beta_{0}$ .

The approaches to handling missing data are assessed by comparing bias in the estimate of the mean, and increase in standard error compared to the true value obtained when data are complete.

We run 100 repetitions. Multiple Imputation using Tobit regression is conducted in STATA and all other aspects of the simulation are conducted in R. We note that MI using Tobit regression is currently not implemented by R packages for MI such as jomo or mice.

#### 5.1.1. Results

**Figure 4.**
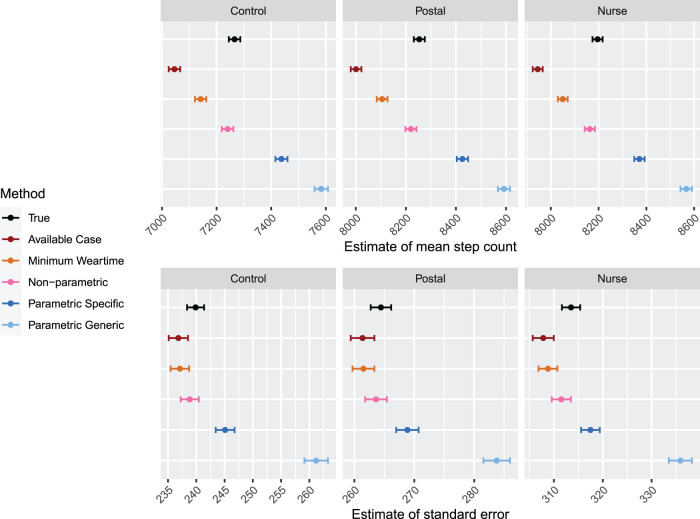
Results for Simulation 1: One measurement period, Scenario A. Results are shown by arm. For each method, estimates for the mean step count are shown in the top panels, and estimates for the standard error of the mean, are shown in the bottom panels. The error bars indicate ±1.96× MC error. Sample size is 120 per arm.

Results of the simulation with Scenario A, where 45% of participants have non-wear and/or sleep extra, are shown in Figure 4. The top panels display the estimates of the mean within each arm. We note that the true value has an MC error associated with it, since a new subsample of complete data is generated in each repetition of the simulation. We observe that available case leads to a downward bias of over 200 steps in all arms. This is expected as the available case assumes that the data with induced missingness is complete. The minimum weartime approach leads to a slight downward bias. The non-parametric approach leads to estimates that are closest to the true value; they are within MC error, but it appears that there is a small downward bias. Both parametric approaches – with the generic and specific upper bound – lead to upward bias, with the generic upper bound in particular leading to an upward bias of over 300 steps in all arms.

The bottom panels of Figure 4 show the estimate of the standard error. The available case approach leads to a slight decrease in SE which is likely due to the fact that the dataset with non-wear and/or sleep extra has a lower mean, and its standard error is generally lower. The minimum weartime approach also leads to a slight decrease in SE. For the non-parametric approach, we observe that the standard error is within the MC error of the true standard error, but appears to be slightly smaller. This is due to the slight downward bias in the estimates of the mean in the non-parametric approach. Finally, we observe that the parametric approaches lead to comparatively larger increases in SE, particularly when a generic upper bound is used.

In Supplemental File 1, we provide central processing unit (CPU) times for running different missing data methods in this simulation.

**Figure 5.**
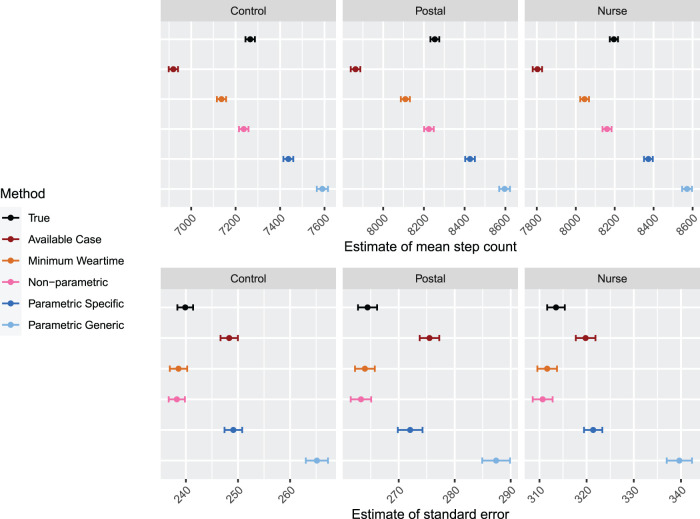
Results for simulation for one measurement period: Scenario B. Results are shown by arm. For each method, estimates for the mean step count are shown in the top panels, and estimates for the standard error of the mean, are shown in the bottom panels. The error bars indicate ±1.96× MC error. Sample size is 120 per arm.

Results of the simulation with Scenario B, where 45% of participants have non-wear and/or sleep extra and an additional 2% of patients have no data for the entire week, are shown in Figure 5. The conclusions are similar to those given for Scenario A, except for one difference. When there are entire weeks that have no data, the available case leads to estimates of the standard error that are much larger than in Scenario A, since there the 2% of patients with no data lead to much greater variability.

### 5.2. Simulation 2: Two measurement periods

The second simulation explores the setting where there is a treatment effect of interest. Data is collected at two measurement periods – baseline and follow-up – and there is missingness in the follow-up data. The aim is to assess the MI approaches in estimating the regression coefficients of a model which regresses the average step count at follow-up on the average step count at baseline and treatment arm. We also compare the MI approaches in estimating the correlation between the average step count between baseline and follow-up within each arm.

In this simulation, we obtain data from the 310 patients in the PACE-UP trial who have complete data at both baseline and 12 months (104 in the control group, 105 in the postal group and 101 in the nurse group). Similarly to the previous simulation, in each repetition of the simulation study, we obtain a bootstrap sample where 85 patients in each treatment group are sampled without replacement. This creates a sample of 255 patients with complete data for each repetition of the simulation.

We then generate missingness under the following two scenarios:

- Scenario A: for a randomly selected 45% of these 255 patients, sleep-extra periods and/or non-wear periods are induced in the 12-month data only by randomly selecting a patient from the PACE-UP trial who has incomplete data at 12 months, and induce sleep-extra and/or non-wear periods according to their missingness pattern.
- Scenario B: In addition to inducing sleep-extra and/or non-wear periods in 45% of patients, a randomly selected 2% of patients provide no data at 12 months.

The following approaches are used to handle the missing data:

- *Minimum weartime approach*: Participants who provide at least 1 day of at least 540 min of weartime at 12 months are included. The daily step count for any day with weartime of less than 540 min is set to missing. The average of the non-missing days are computed at baseline and at 12 months.
- *Non-parametric MI*, as described in Section 4.3. We use BMI, sex, age, average step count at baseline and average weartime at baseline as matching variables where a non-self donor is needed. We set M=10 .
- *Parametric MI*, with specific and generic upper bounds, as described in Section 4.2. We include BMI, sex, age and average step count at baseline as covariates in the imputation model. We set M=10 .

The estimands of interest are the coefficients and standard errors of the following regression model:

(2) $${\bar{y}}_{i,1,.,.}=\beta_{0}+\beta_{1}{\bar{y}}_{i,0,.,.}+\beta_{2}I({\text{arm}}_{i}=\text{postal})+\beta_{3}I({\text{arm}}_{i}=\text{nurse})+\epsilon_{i}$$

where I(⋅) denotes the indicator function. We assume that $e_{i}∼N(0,\sigma^{2})$ .

We assess bias in the estimates of the regression coefficients and increase in the standard error compared to the true value obtained when data are complete. An additional estimand is the correlation of average step count across the week between baseline and 12 months for each arm. Although equation (2) assumes that these correlations are equal across arms, we wish to examine how well the approaches to missing data are able to preserve the correlation within each arm, as correlation is expected to be attenuated.

We run 100 repetitions. MI using Tobit regression is conducted in STATA and all other aspects of the simulation are conducted in R.

#### 5.2.1. Results

**Figure 6.**
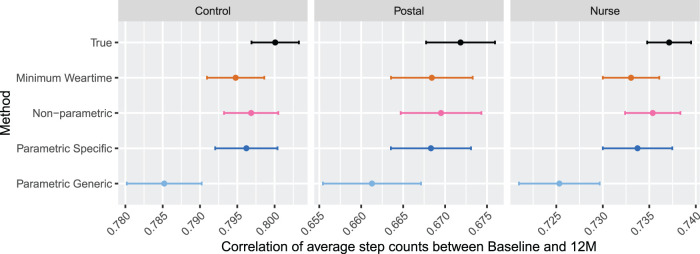
Results for Simulation 2: Two measurement periods, Scenario A. Results are shown by arm. For each method, estimates for correlation between baseline and 12-month average step count is displayed for M=1 . The error bars indicate ±1.96× MC error. Sample size is 85 per arm.

**Figure 7.**
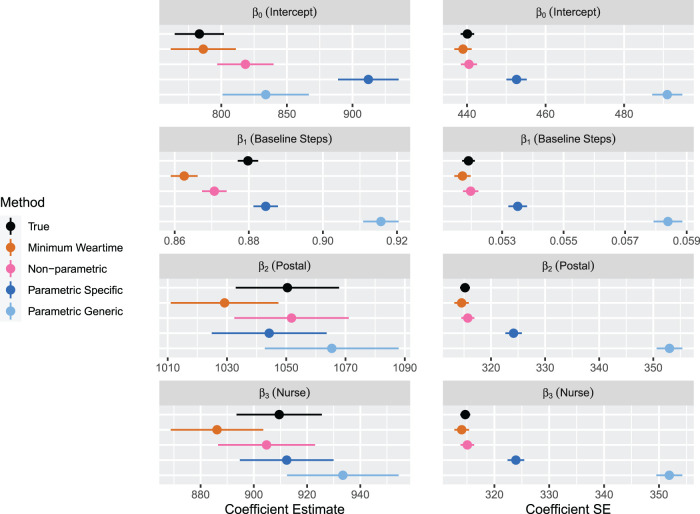
Results for Simulation 2: Two measurement periods, Scenario A. Results are shown by arm. For each method, regression coefficients and standard errors for equation (2) are displayed. The error bars indicate ±1.96× MC error. Sample size is 85 per arm.

Results for simulation with Scenario A, where 45% of participants have non-wear and/or sleep-extra, are displayed in Figures 6 and 7. In Figure 6, the correlation between the average step count between baseline and 12 months are displayed for each arm. We observe that the true correlation is lower in the treatment groups compared to the control group, which is unsurprising in the presence of a treatment effect. We observe that the correlation is attenuated the least compared to the true value for the non-parametric approach. Using a generic upper bound leads to greater attenuation than the specific upper bound when using a parametric approach. The correlations for the minimum weartime approach appears to be comparable to that of the parametric approach with the person-specific upper bound.

In Figure 7, results for estimates of the mean and standard error of the effects of the regression model in equation (2) are shown for Scenario A. The estimates for the intercept produced by the non-parametric and minimum weartime approach are within MC error of the true values; the non-parametric approaches lead to slightly higher estimates. The parametric approaches result in an upward bias of the intercept. For the effect of average stepcount at baseline, the parametric approach with specific upper bound leads to the least biased estimate. The non-parametric approach and minimum weartime approach are downward biased, and the parametric approach with generic upper bound leads to a large upward bias. Of particular interest are the effects of treatment (postal and nurse), which are both estimated well by the non-parametric approach and the parametric approach with specific upper bound. The minimum weartime approach leads to a slightly lower estimate, and the parametric approach with generic upper bound leads to a slightly larger value of estimated effect. Across all coefficients, we observe that the non-parametric approach and minimum weartime approach lead to the smallest standard errors, and the parametric approach with person-specific upper bounds leads to smaller standard errors than the generic upper bounds. Overall, we observe that the non-parametric and parametric approach with person-specific upper bounds provide estimates of the treatment effect that are least biased, and the non-parametric approach additionally provides a smaller standard error.

**Figure 8.**
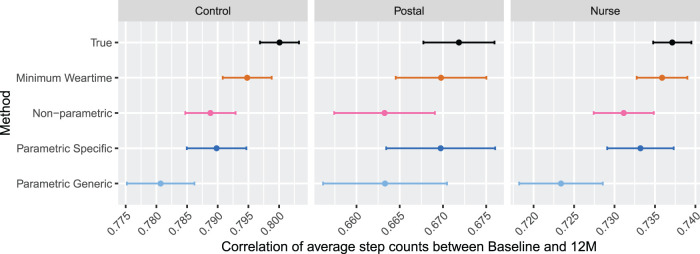
Results for Simulation 2: Two measurement periods, Scenario B. Results are shown by arm. For each method, estimates for correlation between baseline and 12-month average step count is displayed for M=1 . The error bars indicate ±1.96× MC error. Sample size is 85 per arm.

**Figure 9.**
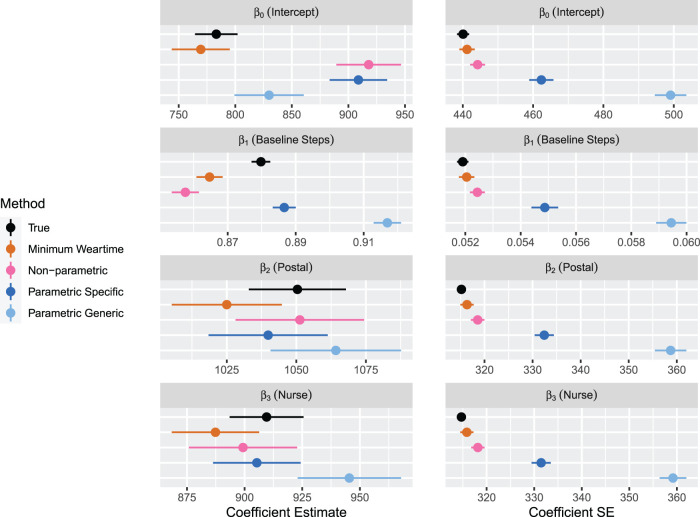
Results for Simulation 2: Two measurement periods, Scenario B. Results are shown by arm. For each method, regression coefficients and standard errors for equation (2) are displayed. The error bars indicate ±1.96× MC error. Sample size is 85 per arm.

Results for the simulation with Scenario B, where 45% of participants have non-wear and/or sleep-extra at 12 Months, and an additional 2% of patients have no data at 12 months, are displayed in Figures 8 and 9. In Figure 8, the correlation between the average step count between baseline and 12 months are displayed for each arm. We observe that the correlation for the non-parametric approach and parametric approach with specific upper bound have a similar level of attenuation for the control and nurse groups; the non-parametric approach leads to slightly more attenuation in the postal group. The parametric approach with the generic upper bound leads to a greater amount of attenuation compared to the other imputation approaches. The minimum weartime approach appears to retain the correlation better than other approaches in this setting. Compared to Scenario A, the non-parametric approach appears slightly less effective at preserving the correlation between baseline and 12 months when there are participants with the entire week missing.

In Figure 9, results for estimates of the mean and standard error of the effects of the regression model in equation (2) are shown for Scenario B. All approaches except the minimum weartime approach lead to upward bias in the estimate of the intercept; the non-parametric approach and parametric approach with specific upper bound lead to a similar amount of bias. For the effect of average stepcount at baseline, the parametric approach with specific upper bound leads to estimates that are closest to the true values. The non-parametric approach and minimum weartime approach lead to a downward bias, while the parametric approach with generic upper bound leads to a large upward bias. The effects of treatment (postal and nurse) are estimated with least bias for the non-parametric approach. The minimum weartime approach leads to a downward bias, while the parametric approach with generic upper bound leads to slightly larger values of estimated effect. We again observe that the that the non-parametric approach and minimum weartime approach lead to the smallest standard errors, and the parametric approach with person-specific upper bounds leads to smaller standard errors than the generic upper bounds. Thus, focusing specifically on the effects of treatment, we observe that the non-parametric approach leads to estimates that are least biased and with smallest standard error.

In the simulations for one measurement period, we observed a very strong advantage for the non-parametric approach compared to other approaches. When there is data from two measurement periods, we still observe that the treatment effects are estimated best by the non-parametric approach; the estimates are least biased, and standard errors are much smaller than those produced by the parametric approach. For the estimates of the intercept and effect of the average baseline stepcount, there is no clear advantage of the non-parametric approach, particularly when whole-week imputation is required. Due to the relatively small sample size used in the simulation, we note that the pool of non-self donors becomes limited to a small number of patients for the non-parametric approach, which may hinder slightly its performance in this setting.

## 6. Application to the PACE-UP trial

We now apply the proposed approaches to handling missingness to an analysis of the PACE-UP trial. In the previous section, missingness was induced in the simulated datasets; we now demonstrate the applicability of the proposed approaches to a genuine trial dataset with real instances of missingness. Table 1 illustrates the breakdown of the total 1023 participants (338 in usual care, 339 in the postal group and 346 in the nurse group) into the types of missingness observed. We classify participants by whether they have completely observed data, non-wear periods only, sleep-extra only, or both non-wear and sleep-extra. Participants may also have more than 5 days with weartime <300 minutes in which case the entire week is imputed in the non-parametric approach. These participants are excluded in the minimum weartime approach. We also illustrate the proportion of participants that have zero days with weartime <540 minutes between 1 and 5 days (inclusive), and greater than 5 days. There is a greater amount of missingness at 12 months compared to baseline.

**Table 1.** The number of patients with each of the missing types and their percentages are shown for PACE-UP trial data at baseline and 12 months.

	Baseline	12 months
**Type of missingness**		
Completely observed	554 (54.2%)	473 (46.2%)
Non-wear only	361 (35.3%)	287 (28.1%)
Sleep-extra only	51 (4.99%)	104 (10.2%)
Non-wear and sleep-extra	57 (5.57%)	94 (9.19%)
Whole week imputation	0 (0%)	65 (6.35%)
**Number of days with weartime <540 min**	
0	696 (68.0%)	562 (54.9%)
Between 1 and 5	327 (32.0%)	396 (38.7%)
Greater than 5	0 (0%)	65 (6.35%)
Total	1023	1023	

*Note:* There are no cases of whole week imputation or days of weartime <540 min at baseline by design, via trial inclusion criteria.

We analyse the data using a linear model which regresses the average step count at 12 months on the average step count at baseline, arm and primary care practice:

(3) $$\begin{array}{rl} {\bar{y}}_{i,2,...} & =\beta_{0}+\beta_{1}{\bar{y}}_{i,0,...}+\beta_{2}I\left({\text{arm}}_{i}=\text{postal}\right)\\ & \;+\beta_{3}I\left({\text{arm}}_{i}=\text{nurse}\right)+\beta_{4}P2_{i}+\beta_{5}P3_{i}+\cdots +\beta_{9}P7_{i}+\epsilon_{i} \end{array}$$

where P2,P3,…,P7 are dummy variables for the primary care practice that the participant is registered at. We assume that $e_{i}∼N(0,\sigma^{2})$ .

We note that this is a simpler analysis compared to the primary analysis of the PACE-UP trial, where additional covariates (sex and age group) were included, and a clustering effect was included to account for household, since a small number of participants were in couples. We have removed some of the complexities of the original analysis in order to focus on the comparison between different approaches to handle the missing data.

The following methods of handling missing data are considered:

- *Minimum weartime approach*: Participants who provide at least 1 day of at least 540 minutes of weartime at 12 months are included. The daily step count for any day with less than 540 minutes of weartime is set to missing. The average of the non-missing days are computed at baseline and at 12 months. We use the weartime calculated by the Actilife software.
- *Non-parametric MI*, as described in Section 4.3. Imputation is conducted firstly in the baseline dataset, separately in the three treatment arms. We use BMI, sex and age as matching variables where a non-self donor is needed. The average of the imputed baseline average stepcounts are computed, which is then used for the imputation at 12 months, which is again conducted separately in the three treatment arms. At 12 months, we use BMI, sex, age, average step count at baseline and average weartime at baseline as matching variables where a non-self donor is needed.
- *Parametric MI*, with specific and generic upper bounds, as described in Section 4.2. The seven days at baseline, and seven days at 12 months are modelled as jointly normally distributed, conditional on covariates BMI, sex, and age. Imputation is performed separately in each arm.

**Figure 10.**
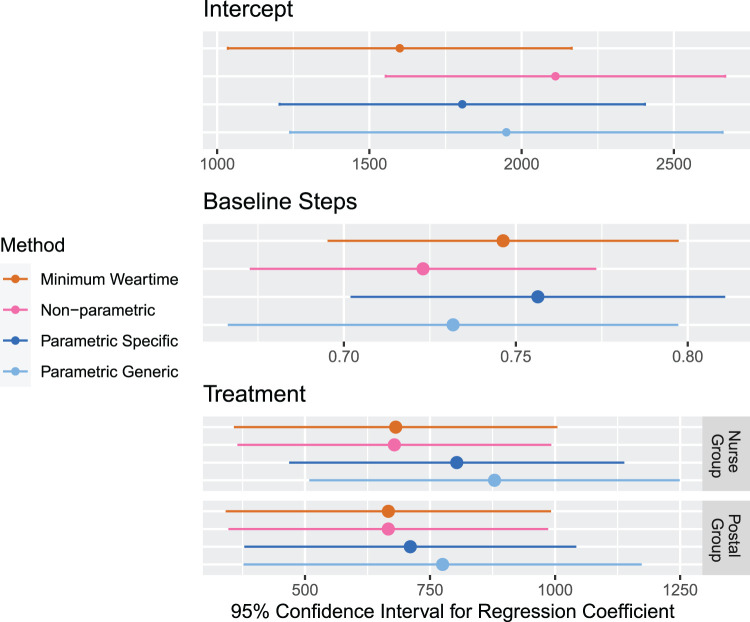
Results for the analysis of the PACE-UP trial. For each method of handling missing data, the 95% confidence intervals for the effects for the regression model in equation (3) is displayed. The 95% confidence intervals for the effects of practices are shown in Figure 14 in the Appendix.

Results for the estimated effects effects and their estimated confidence intervals produced by each method are displayed in Figure 10. The means and standard errors of the effects are displayed in Table 2. The confidence intervals across the methods overlap, but we observe noticeable differences in the point estimates and standard errors which reveal the potential impact that missing data assumptions can have on the results of the primary analysis.

**Table 2.** Results for the analysis of the PACE-UP trial. For each method of handling missing data, the estimated means (with standard errors in parentheses) are shown for the effects for the regression model in equation (3) is displayed.

	Estimated means and standard errors			
Coefficient	Minimum weartime	Non-parametric	Parametric specific	Parametric generic
Intercept	1599.65 (288.05)	2110.96 (284.29)	1804.87 (305.98)	1949.72 (362.10)
Baseline steps	0.75 (0.03)	0.72 (0.03)	0.76 (0.03)	0.73 (0.03)
Postal group	666.69 (166.00)	666.49 (163.12)	710.55 (169.37)	775.10 (203.06)
Nurse group	681.35 (165.05)	678.65 (160.16)	803.41 (170.95)	878.96 (188.94)
P2	−831.83 (266.32)	−658.93 (255.24)	−581.28 (282.59)	−422.90 (321.30)
P3	−287.51 (256.00)	−446.34 (248.35)	−395.47 (269.78)	−312.76 (293.50)
P4	−658.03 (280.20)	−511.25 (269.67)	−369.16 (293.85)	−348.26 (327.41)
P5	35.25 (248.72)	184.13 (240.16)	194.49 (258.25)	337.40 (299.67)
P6	78.29 (265.13)	136.54 (257.50)	165.02 (291.26)	325.56 (316.06)
P7	−65.11 (332.66)	110.05 (324.23)	−18.34 (341.61)	12.28 (379.22)

Note that the practices have been included to reflect the design of the study, but their coefficients should not be the focus of the interpretation.

In the point estimates, we find patterns that are similar to what we observed in Section 5.2. For the intercept, the minimum weartime approach leads to the smallest estimate, and the non-parametric approach leads to the highest estimate. When comparing the coefficients for the average baseline stepcount, it should be noted that the values of the average baseline stepcount are computed using the imputed values at baseline for the non-parametric and parametric approaches, so their values are different across approaches. In the effects for treatment, we observe that the minimum weartime and non-parametric approach leads to the smallest point estimates, and the parametric approaches lead to the largest estimates.

Across all coefficients, we find that the standard errors are smallest for the non-parametric and minimum weartime approaches; the non-parametric approach leads to slightly smaller standard errors. The parametric approaches produce larger standard errors; the generic upper bound, in particular, leads to the largest standard errors. These observations are consistent with what we found in the simulation studies.

**Figure 15.**
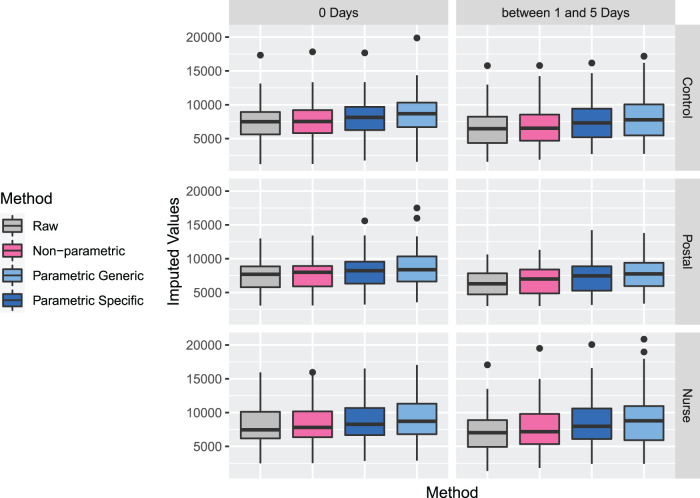
Boxplots compare the raw value with the imputed values at baseline under the non-parametric and parametric approaches when there are zero days with weartime <540 min (left panel) and between 1 and 5 days with weartime <540 min (right panel). Results are shown by treatment arm.

**Figure 16.**
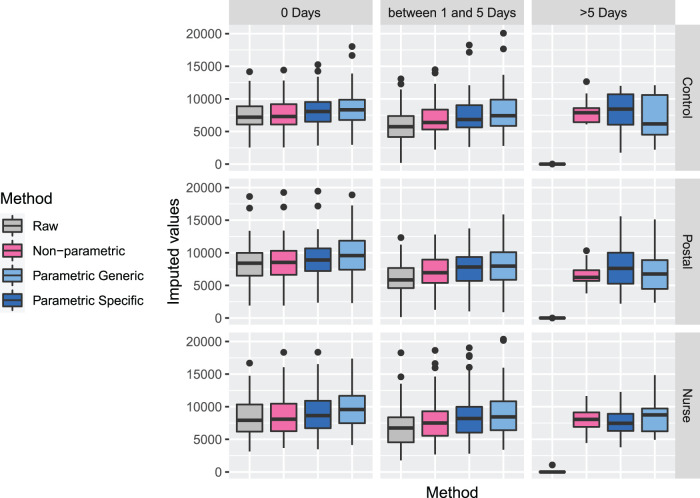
Boxplots compare the raw value with the imputed values at 12 months under the non-parametric and parametric approaches when there are zero days with weartime <540 min (left panel) and between 1 and 5 days with weartime <540 min (middle panel) and more than 5 days with weartime <540 min (right panel). Results are shown by treatment group.

Figure 15 in the Appendix displays boxplots comparing the raw values with the imputed values for M=1 for each treatment group at baseline. We display the boxplots separately by the extent of missingness, classifying participating as having zero days with weartime of less than 540 minutes or between 1 and 5 days with weartime of less than 540 minutes. We observe that the non-parametric approach leads to a slightly larger values compared to the raw values, the parametric approach with specific upper bound leads to slightly higher values than that. The parametric approach with generic upper bound, which does not use the epoch-level information on missingness, leads to the highest imputed values. In Figure 16, we observe the equivalent boxplots for 12 months, where there is an additional column for participants where the entire week is missing. These participants are excluded in the minimum weartime approach. In this column, we find that distributions have more skewness. In particular, the non-parametric approach, which imputes the whole week with non-self donors, leads to very small variability in imputed values compared to the parametric approaches.

The original analysis of the PACE-UP trial reported an estimated effect of the postal intervention of 642 (with standard error 160) and effect of the nurse intervention of 677 (with standard error of 159) when using a minimum weartime approach.^2^ Their imputation analysis which includes participants with missing data at 12 months uses a day-level imputation model with the following variables: treatment group, baseline steps, gender, age, practice, and month of baseline accelerometry. This analysis produced an estimated effect of the postal intervention of 638 (with standard error 160) and effect of the nurse intervention of 679 (with standard error of 159). While a precise comparison with our re-analysis cannot be made since the analysis models differ, we note that the estimates for the effects of treatment produced by the epoch-level non-parametric approach are most similar to those produced in the original study.

The purpose of this analysis is to compare the impact of the differing approaches to missing data on the model results. We have therefore kept the analysis and imputation models relatively simple, and assume that the data are MAR given the observed data. A full analysis should include sensitivity analyses to the MAR assumption. This would involve careful consideration of appropriate departures from the MAR assumption. For example, one might consider the following scenarios:

- If participants take the accelerometer off too early in the evening, or put it on late in the morning, it may be because they are at home and not being particularly active. Under this assumption, we would impute values that are lower than that assumed under MAR.
- If participants did not provide sufficient weartime for more than 5 days a week (which would lead to whole-week imputation in the non-parametric approach), this may be because the participant is less active than usual during the week and does not feel motivated to wear the device. Under this assumption, we would impute values that are lower than that assumed under MAR.
- Some participants may remove the device while they are exercising as they find it uncomfortable to wear. In this case, it is possible that values above that assumed under MAR should be imputed. However, identifying periods where participants removed the device for this reason is difficult to discern without information from, for example, activity reports from participants.

Further, in addition to considering departures from the MAR assumption, a full analysis should take into account additional auxiliary variables in the imputation model, such as weather variables, which have shown to be predictive of daily step count and also of whether daily step count is missing.^6^

## 7. Discussion

This paper described the challenges of applying MI to epoch-level accelerometer data; namely, the difficulty in identifying and classifying missingness, and the complicated nature of epoch-level distributions. Possible methods of overcoming these challenges are presented. Firstly, a novel approach to classifying epoch-level zero-count periods into inactive, non-wear, sleep and sleep-extra periods is presented, which carefully teases out differences between per-protocol instances of no activity, short periods of inactivity which are not missing data, and periods where missing data is incurred due to participants removing the device. These missing periods can be handled with either parametric and non-parametric MI. In the parametric approach, step counts on the day-level are imputed using epoch-level information on the missing periods per day to determine a person-specific upper bound for Tobit regression. In the non-parametric approach, missing periods are replaced by self- or non-self donors.

These approaches were compared using simulations where zero-count periods are generated using missingness patterns observed in the dataset. Simulation studies conducted in the literature to date often induce missingness in hour-long chunks, which does not reflect the complexities of how missingness arises in practice in the accelerometer setting. The simulations showed the merits of using the epoch-level information in MI. In the setting where the average step count for each treatment group is estimated for one measurement period, the non-parametric approach leads to estimates with the least bias and highest precision. The parametric approach with the specific upper bound leads to less bias and more precision than the parametric approach with the generic upper bound. Where data has been collected over two measurement periods and the analysis model regresses the average step count at 12 months on the average step count at baseline and treatment group, the non-parametric approach and parametric approach with specific upper bound have a more comparable performance, but the non-parametric approach leads to least biased point estimates for the treatment effects while maintaining a small standard error. By considering settings where there is data for just one measurement period as well as two measurement periods, these findings are relevant to cross-sectional as well as longitudinal analyses of accelerometer data.

We performed a re-analysis of the 2017 PACE-UP trial, where the results for a simplified analysis model using the minimum weartime approach, the non-parametric approach, and parametric approaches with specific and generic upper bounds are compared. While the approach to missingness does not overall change conclusions of the study, they point to potentially important implications for results. In particular, we observe that estimated effects of treatment are slightly higher for the parametric approaches, and the standard errors are larger for the parametric approaches, mirroring results from the simulations. In the original analysis of the PACE-UP trial, Harris et al.^2^ conducted sensitivity analyses on the impact of using different thresholds on weartime for defining missingness (e.g. requiring a minimum of 600 minutes of weartime for a complete day, compared to 540 minutes in the main analyses). Our analysis additionally reveals the impact of taking an epoch-level perspective on missing data.

There are a number of important avenues for further work. Firstly, as discussed in Section 6, sensitivity analyses for the MAR assumption are an important consideration in the context of these epoch-level approaches to MI. As participants may be likely to remove their accelerometer during inactive periods, considering the implication of the data being MNAR is important. Secondly, it is worth exploring potential adaptations to the non-parametric approach to improve its performance in the setting where there are entire weeks of data missing. Our simulations studies indicate that replacing entire weeks of data with non-self donors appears to reduce the performance of this approach, potentially because the number of available donors are insufficient in this setting. A hybrid approach which imputes non-wear and sleep-extra non-parametrically and takes advantage of the parametric approach for entire missing weeks would be a potential solution. Thirdly, we have considered the setting where whole-week imputation occurs only at 12 months, which was the case in the PACE-UP trial. In other studies, there may be participants with missing weeks at baseline included in the study, in which case, donors for whole-week imputation at baseline may be determined by physical activity characteristics obtained at 12 months. For an extended analysis of the PACE-UP trial including outcomes at 3 months and 3 years in addition to baseline and 12 months, an approach to identifying suitable donors in this more complex setting with multiple measurement periods will need to be identified.

Applying these methods for handling missing data in large observational studies will be computationally intensive, as there may be thousands of individuals and months or years of data from each individual. In these settings, it would be sensible to split up the measurement periods into sections, so that the classification algorithm is implemented within each section separately. The imputation process can be completed with parallelization to reduce computational time.

While our study has focused on accelerometer studies, there is a growing need to consider missingness at a finer epoch-level for a number of health outcomes in trials, particularly as continuous monitoring participants through digital devices become more common.^20^ For example, studies that remotely monitor vital signs of people with dementia use a number of sensor and wearable devices that track data in fine intervals of time.^21^ In applications to other devices, there are key differences to consider in the classification algorithm. For example, wearables that measure additional outcomes such as heartrate would be able distinguish between inactivity and missing data, and waterproof wearables should, in principle, not lead to non-wear due to showering or swimming. The multiple imputation approaches will be broadly applicable, regardless of the type of device used. Adapting the classification algorithm to extend its applicability for other digital devices is an important direction for future research.

## Supplemental Material

sj-pdf-1-smm-10.1177_09622802231188518 - Supplemental material for Multiple imputation approaches for epoch-level accelerometer data in trials Supplemental material, sj-pdf-1-smm-10.1177_09622802231188518 for Multiple imputation approaches for epoch-level accelerometer data in trials by Mia S Tackney, Elizabeth Williamson, Derek G Cook, Elizabeth Limb, Tess Harris and James Carpenter in Statistical Methods in Medical Research

sj-zip-2-smm-10.1177_09622802231188518 - Supplemental material for Multiple imputation approaches for epoch-level accelerometer data in trials Supplemental material, sj-zip-2-smm-10.1177_09622802231188518 for Multiple imputation approaches for epoch-level accelerometer data in trials by Mia S Tackney, Elizabeth Williamson, Derek G Cook, Elizabeth Limb, Tess Harris and James Carpenter in Statistical Methods in Medical Research

## Figures and Tables

**Figure 11.**
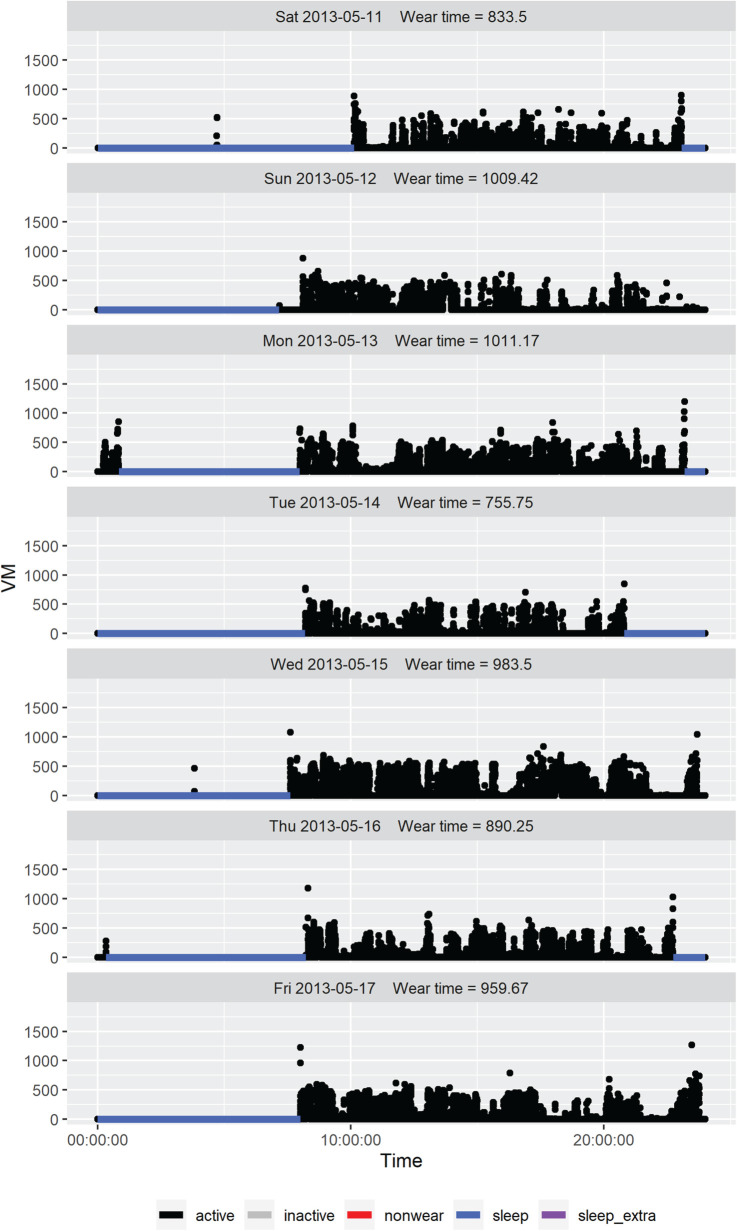
An example of 7 days of accelerometer data, where vector magnitude at the epoch-level is plotted against time. No missing data is detected.

**Figure 12.**
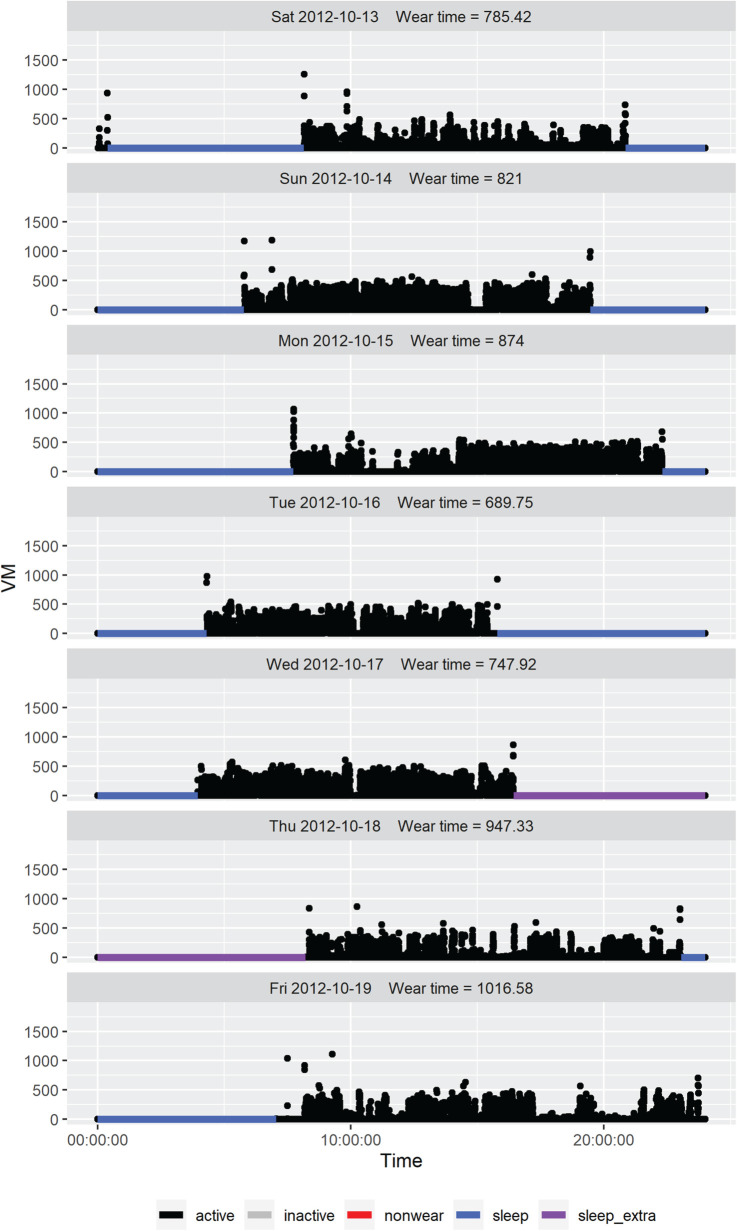
An example of 7 days of accelerometer data, where vector magnitude at the epoch-level is plotted against time. Sleep-extra is detected between Wednesday and Thursday.

**Figure 13.**
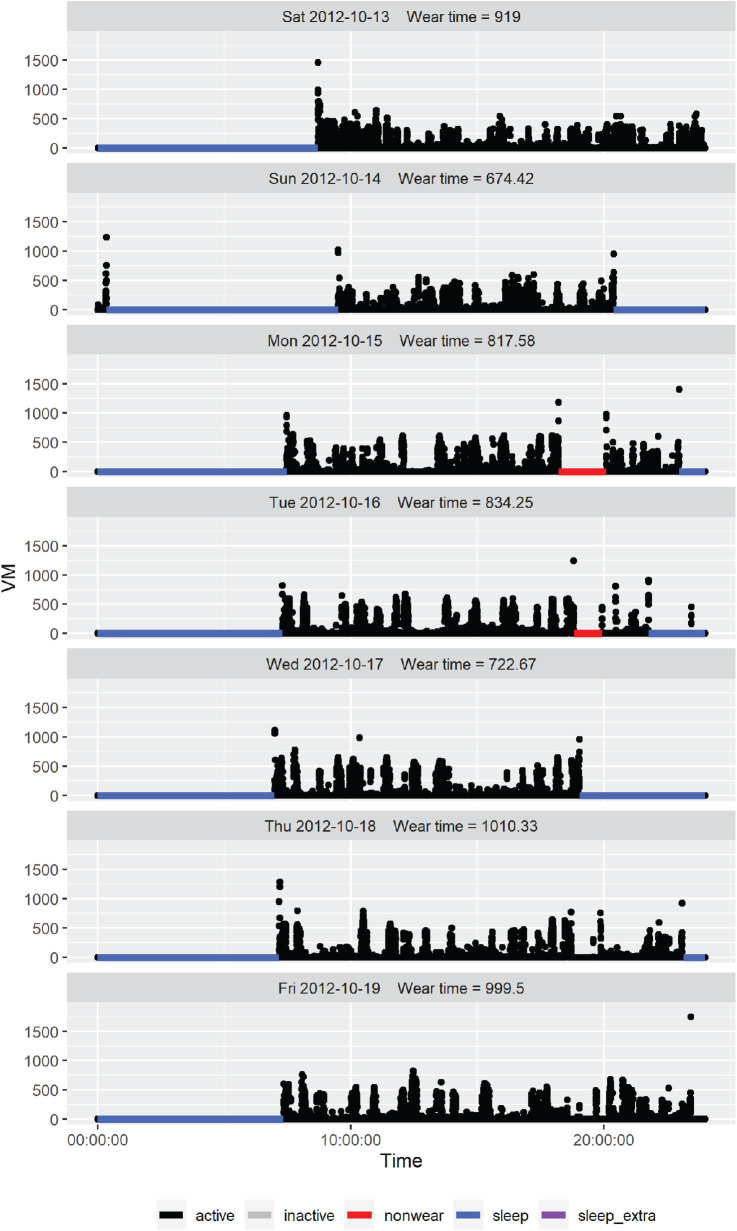
An example of 7 days of accelerometer data, where vector magnitude at the epoch-level is plotted against time. Non-wear periods are detected on Monday and Tuesday.

**Figure 14.**
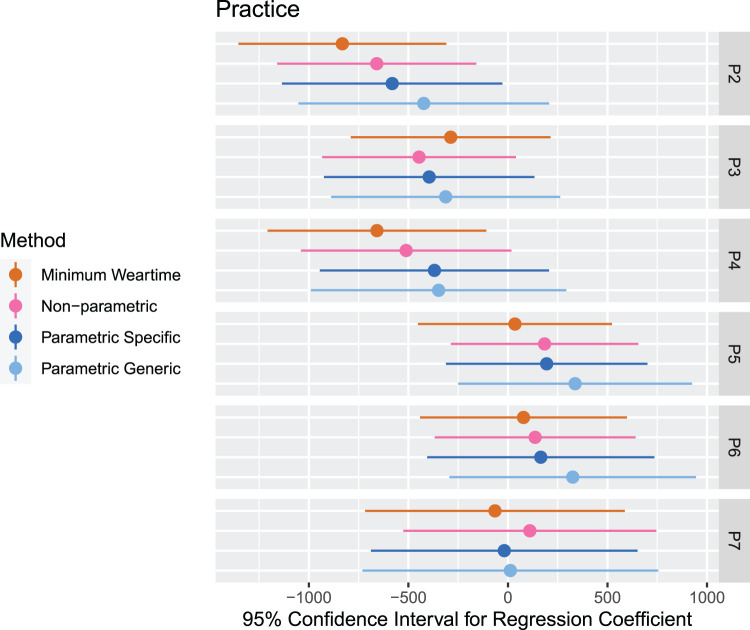
Results for the analysis of the PACE-UP trial. For each method of handling missing data, the 95% confidence intervals for the effects of practices are displayed.

## Appendix

### Plots of vector magnitude at the epoch-level

See Figures 11 to 13.

### PACE-UP trial analysis: Further results

See Figures 14 to 16.
