# Supplementary material for: Multiple imputation approaches for epoch-level accelerometer data in trials
Source: Stat Methods Med Res. 2023 Jul 31;32(10):1936–60. doi: 10.1177/09622802231188518 (PMC10563375; doi:10.1177/09622802231188518)
Supplement: sj-pdf-1-smm-10.1177_09622802231188518 - Supplemental material for Multiple imputation approaches for epoch-level accelerometer data in trials [file sj-pdf-1-smm-10.1177_09622802231188518.pdf]

Multiple Imputation Approaches for Epoch-level Accelerometer data in Trials: Supplementary File 1

For illustrative purposes, we compare the central processing unit (CPU) time for different approaches to handling missing data in the setting of Simulation 1: Scenario A, where there are 120 participants in each of the three trial arms, and 45% of participants have sleep-extra and/or nonwear periods. We consider a single repetition of the simulation study.

Table 1 displays CPU time in seconds for taking day-level approaches. Here, epoch-level data are aggregated at the day level to obtain daily step counts, and analyses are conducted on the day-level data. There is no classification of missingness or imputation at the epoch-level.

Table 1: CPU time in seconds for running day-level approaches to handling missing data for the setting in Simulation 1: Scenario A. Analyses were run on a Windows machine with a 64-bit processor and 16 GB of memory.

**Missing Data Approach**	**CPU time (seconds)**
Minimum Weartime	0.6
Available Case Analysis	0.6
Parametric MI: Generic	102.2

Table 2 displays CPU time in seconds for epoch-level approaches. All epoch-level approaches first classify intervals into active, inactive, nonwear, sleep and sleep-extra periods. In the chosen dataset, this took 17.3 seconds. We then compute the CPU time taken for each each approach to handling missing data. The total CPU time is the time taken for the classification and the handling of missing data.

Table 2: CPU time in seconds for running epoch-level approaches to handling missing data for the setting in Simulation 1: Scenario A. Analyses were run on a Windows machine with a 64-bit processor and 16 GB of memory.

**Missing Data Approach**	CPU time for classifying missing data	CPU time for handling missing data	Total CPU time
**Minimum Weartime**	17.3	0.6	17.9
**Available case Analysis**	17.3	0.6	17.9
**Non-parametric MI**	17.3	41.8	59.2
**Parametric MI: Specific**	17.3	101.2	118.5
**Parametric MI: Generic**	17.3	92.8	110.1
