# Supplementary material for: Multiple imputation approaches for epoch-level accelerometer data in trials
Source: Stat Methods Med Res. 2023 Jul 31;32(10):1936–60. doi: 10.1177/09622802231188518 (PMC10563375; doi:10.1177/09622802231188518)
Supplement: sj-zip-2-smm-10.1177_09622802231188518 - Supplemental material for Multiple imputation approaches for epoch-level accelerometer data in trials [file sj-zip-2-smm-10.1177_09622802231188518.zip › Vignette.html]

Vignette


 


# Vignette for Non-Parametric Imputation of Epoch-level Accelerometer Data¶

## Supplementary Materials¶

#### Author: Mia S. Tackney¶

#### Date: 6 December 2022¶

## Introduction¶

This vignette introduces a suite of R functions which handle epoch-level data from the GT3X+ accelerometer.

These accelerometers have been used in a number of clinical trials to measure participants' physical activity over the course of a week, before and after an intervention.

Specifically, functions introduced in this vignette use data on Vector Magnitude (VM) and Step count measured at every epoch (usually set at 5 second intervals) for each participant, and perform the following tasks:

- Analyze common missing data patterns in epoch-level accelerometer data;
- Visualize epoch-level accelerometer data across the measurement period (typically one week);
- Perform non-parametric imputation of missing accelerometer data.

## Setting up and loading data¶

We first install relevant R packages and source files which contain the functions.

In [1]:

```
options(warn=-1)
source("00_init.R", echo=F)
source("03_profile_plot_functions_week.R")
source("05_nonparametric_impute_incomp.R")
source("05_nonparametric_impute_nonself_day_functions_helper.R")
```

```
Attaching package: 'gridExtra'

The following object is masked from 'package:dplyr':

    combine


Attaching package: 'glue'

The following object is masked from 'package:dplyr':

    collapse


Please cite as: 

 Hlavac, Marek (2018). stargazer: Well-Formatted Regression and Summary Statistics Tables.
 R package version 5.2.2. https://CRAN.R-project.org/package=stargazer 


Attaching package: 'lubridate'

The following object is masked from 'package:hms':

    hms

The following objects are masked from 'package:base':

    date, intersect, setdiff, union

*** This is beta software. Please report any bugs!
*** See the NEWS file for recent changes.

Attaching package: 'reshape2'

The following object is masked from 'package:tidyr':

    smiths
```

We load a dataset which contains simulated data based on a small portion of the PACE-UP trial epoch-level dataset. There are 52 patients in this dataset, each providing seven days' worth of data from their accelerometer. Each row of this dataset represents a 5-second epoch where vector magnitude and step count is measured by the accelerometer. This dataset contains the following variables:

- Patient ID number
- Date
- Day of week
- Day order
- Time at the start of the epoch
- Step count for the duration of the epoch
- Vector magnitude (VM) for the duration of the epoch
- Wear time per day
- Gender
- Age
- BMI at baseline

The values for patient ID number, Gender, Age and BMI at baseline are fabricated, so these data are not identifiable. These data are extracted from GT3X+ accelerometer using the Actilife Software. Typically, a large number of other variables can be extracted such as time spent in different types of activities.

In [16]:

```
Example <- readRDS("Exampledat.RDS")
head(Example)
```

| Age | Gender | Date | Day.of.Week | Day\_order | Steps.Counts | Weartime\_R | Time | Steps | VM | bmib | ID |
| --- | --- | --- | --- | --- | --- | --- | --- | --- | --- | --- | --- |
| 55 | F | 2014-01-23 | Thursday | 1 | 5950 | 914.5833 | 00:01:00 | 0 | 0 | 27.49702 | 52 |
| 55 | F | 2014-01-23 | Thursday | 1 | 5950 | 914.5833 | 00:01:05 | 0 | 0 | 27.49702 | 52 |
| 55 | F | 2014-01-23 | Thursday | 1 | 5950 | 914.5833 | 00:01:10 | 0 | 0 | 27.49702 | 52 |
| 55 | F | 2014-01-23 | Thursday | 1 | 5950 | 914.5833 | 00:01:15 | 0 | 0 | 27.49702 | 52 |
| 55 | F | 2014-01-23 | Thursday | 1 | 5950 | 914.5833 | 00:01:20 | 0 | 0 | 27.49702 | 52 |
| 55 | F | 2014-01-23 | Thursday | 1 | 5950 | 914.5833 | 00:01:25 | 0 | 0 | 27.49702 | 52 |

## Analysing epoch-level data¶

### Classifying Runs¶

The calc\_runs() classifies epoch-level data into the following possible activity categories: active, sleep, inactive, nonwear and sleep-extra, and identifies the start and end times of each activity. The output provides:

- start: the epoch number when the activity starts
- end: the epoch number when the activity ends
- length: the length, in minutes, of the activity
- type: the type of activity, which can be active, sleep, inactive, nonwear, sleep-extra
- start\_date: the date of the start of activity
- start\_time: the time of the start of activity
- end\_date: the date of the end of the activity
- end\_time: the time of the end of the activity

We demonstrate for patient 52 the activites across their 7 days.

In [12]:

```
calc_runs(Example %>% filter(ID=="52"))
```

1. | start | end | length | type | start\_date | start\_time | end\_date | end\_time |
   | --- | --- | --- | --- | --- | --- | --- | --- |
   | 1 | 4796 | 399.66667 | sleep | 2014-01-23 | 00:01:00 | 2014-01-23 | 06:40:35 |
   | 4797 | 15759 | 913.66667 | active | 2014-01-23 | 06:40:40 | 2014-01-23 | 21:54:10 |
   | 15760 | 21984 | 518.75000 | sleep | 2014-01-23 | 21:54:15 | 2014-01-24 | 06:32:55 |
   | 21985 | 32047 | 838.66667 | active | 2014-01-24 | 06:33:00 | 2014-01-24 | 20:31:30 |
   | 32048 | 39603 | 629.66667 | sleep | 2014-01-24 | 20:31:35 | 2014-01-25 | 07:01:10 |
   | 39604 | 52579 | 1081.41667 | active | 2014-01-25 | 07:01:15 | 2014-01-26 | 01:02:30 |
   | 52580 | 58257 | 473.16667 | sleep | 2014-01-26 | 01:02:35 | 2014-01-26 | 08:55:40 |
   | 58258 | 59462 | 100.50000 | active | 2014-01-26 | 08:55:45 | 2014-01-26 | 10:36:05 |
   | 59463 | 60581 | 93.25000 | nonwear | 2014-01-26 | 10:36:10 | 2014-01-26 | 12:09:20 |
   | 60582 | 67329 | 562.41667 | active | 2014-01-26 | 12:09:25 | 2014-01-26 | 21:31:40 |
   | 67330 | 73807 | 539.83333 | sleep | 2014-01-26 | 21:31:45 | 2014-01-27 | 06:31:30 |
   | 73808 | 79056 | 437.50000 | active | 2014-01-27 | 06:31:35 | 2014-01-27 | 13:48:55 |
   | 79057 | 80222 | 97.16667 | nonwear | 2014-01-27 | 13:49:00 | 2014-01-27 | 15:26:05 |
   | 80223 | 85048 | 402.25000 | active | 2014-01-27 | 15:26:10 | 2014-01-27 | 22:08:15 |
   | 85049 | 91000 | 496.00000 | sleep | 2014-01-27 | 22:08:20 | 2014-01-28 | 06:24:15 |
   | 91001 | 102766 | 980.58333 | active | 2014-01-28 | 06:24:20 | 2014-01-28 | 22:44:45 |
   | 102767 | 108369 | 466.91667 | sleep | 2014-01-28 | 22:44:50 | 2014-01-29 | 06:31:40 |
   | 108370 | 113678 | 442.50000 | active | 2014-01-29 | 06:31:45 | 2014-01-29 | 13:54:05 |
   | 113679 | 115258 | 131.66667 | nonwear | 2014-01-29 | 13:54:10 | 2014-01-29 | 16:05:45 |
   | 115259 | 119209 | 329.33333 | active | 2014-01-29 | 16:05:50 | 2014-01-29 | 21:35:00 |
   | 119210 | 120948 | 144.91667 | sleep | 2014-01-29 | 21:35:05 | 2014-01-29 | 23:59:55 |

### Calculating Weartime¶

The calc\_weartime() function calculates the weartime for a specific day. The VM of the day is needed as input. For example, for Patient 52, on their first day using the accelerometer (a Thursday), their weartime is calculated as:

In [13]:

```
P52_Thurs <- Example %>% filter(ID==52 & Day.of.Week=="Thursday")
calc_weartime(P52_Thurs$VM)
```

914.583333333333

We compute weartime for each patient, for each day of wear, and add it as an additional variable in the dataset.

In [19]:

```
Weartime <- Example %>% group_by(ID, Date) %>% summarise(Weartime_R=calc_weartime(VM))
head(Weartime)
Example <- left_join(Example, Weartime, by=c("ID", "Date"))
```

| ID | Date | Weartime\_R |
| --- | --- | --- |
| 1 | 2014-06-07 | 722.1667 |
| 1 | 2014-06-08 | 891.8333 |
| 1 | 2014-06-09 | 756.4167 |
| 1 | 2014-06-10 | 763.3333 |
| 1 | 2014-06-11 | 803.5000 |
| 1 | 2014-06-12 | 775.0833 |

## Visualizing Epoch-level Data¶

The plot\_week() function takes Epoch-level data for a particular patient and plots the Vector Magnitude across each day, and indicates the different activities throughout the day.

In [17]:

```
options(repr.plot.width=5, repr.plot.height=6)
Person1 <- Example %>% filter(ID=="34")
Person2 <- Example %>% filter(ID=="52")
p1 <- plot_week(Person1)
p1
```

In [18]:

```
p2 <- plot_week(Person2)
p2
```

# Non-parametric imputation¶

The impute\_and\_analyse() function carries out non-parametric imputation. In summary, this function carries out the following tasks:

- Identifies missing periods in the dataset, including nonwear, sleep-extra and whole weeks which need to be imputed.
- Peforms multiple imputation either using self or non-self donation. By default, if there are less than four donors in the donor pool, non-self donation is carried out.
- Exports the following files:
  > 1. a file with MM copies of the dataset with the total step counts per week for each patient ID. Where there is missingness during the week the MM values are different. If there is no missingness, the MM values are the same.
  > 2. a file with generic and specific upper bounds for daily step counts for each patient ID, which could be used in STATA to do Tobit regression.
  > 3. a file with missingness patterns saved for each patient ID, which can be used for further analyses.
- Analyses the data by calculating the mean and standard error of the week-average step counts when non-parametric imputation is used, and when missingness is ignored (available case analysis).

Inputs for this function is as follows:

- **Simdata**: dataset containing epoch-level data.
- **sim**: integer for simulation number. Set to 1 by default.
- **M**: integer greater than 1 for the number of imputations.
- **passive**: function used to transform daily step counts due to skewness. Default is log transformation.
- **path**: path to save upperbounds, which can be used to do parametric imputation in STATA.
- **imputations\_save\_path**: path to save all imputations for all M. This can be used to analyse data using more complex models.
- **missing\_save\_path**: path to save missingness classification.
- **missing\_save\_path**: path to save results for complete case analysis if complete case analysis is performed.
- **adj\_base**: set to NULL if average baseline step count is NOT used as a matching variable (default). set to TRUE if used as a matching variables.
- **output**: by default set to TRUE if all output is to be shown. Otherwise set to FALSE.

In [21]:

```
result <- impute_and_analyse(Simdata=Example, sim=1, M=10, passive=log, 
                   path=NULL, imputations_save_path=NULL, missing_save_path=NULL,  cc_save_path=NULL,
                               adj_base=NULL, output=FALSE)
result
```

| sim | Method | mean | se |
| --- | --- | --- | --- |
| 1 | Ignore | 6697.374 | 396.4339 |
| 1 | Complete Case | NA | NA |
| 1 | Non-para | 6978.915 | 372.0837 |

In [ ]:

```

```
