# Supplementary material for: Multiple imputation approaches for epoch-level accelerometer data in trials
Source: Stat Methods Med Res. 2023 Jul 31;32(10):1936–60. doi: 10.1177/09622802231188518 (PMC10563375; doi:10.1177/09622802231188518)
Supplement: sj-zip-2-smm-10.1177_09622802231188518 - Supplemental material for Multiple imputation approaches for epoch-level accelerometer data in trials [file sj-zip-2-smm-10.1177_09622802231188518.zip › Vignette.pdf]

Vignette for Non-Parametric Imputation of Epoch-level Accelerometer Data

Supplementary Materials

Author: Mia S. Tackney

Date: 6 December 2022

Introduction

This vignette introduces a suite of R functions which handle epoch-level data from the GT3X+ accelerometer (https://actigraphcorp.com/support/activity-monitors/gt3xplus/).

These accelerometers have been used in a number of clinical trials to measure participants' physical activity over the course of a week, before and after an intervention.

Specifically, functions introduced in this vignette use data on Vector Magnitude (VM) and Step count measured at every epoch (usually set at 5 second intervals) for each participant, and perform the following tasks:

- Analyze common missing data patterns in epoch-level accelerometer data;
- Visualize epoch-level accelerometer data across the measurement period (typically one week);
- Perform non-parametric imputation of missing accelerometer data.

Setting up and loading data

We first install relevant R packages and source files which contain the functions.

In [1]:

```
options(warn=-1)
source("00_init.R", echo=F)
source("03_profile_plot_functions_week.R")
source("05_nonparametric_impute_incomp.R")
source("05_nonparametric_impute_nonself_day_functions_helper.R")
```

```
Attaching package: 'gridExtra'

The following object is masked from 'package:dplyr':

    combine


Attaching package: 'glue'

The following object is masked from 'package:dplyr':

    collapse


Please cite as:

 Hlavac, Marek (2018). stargazer: Well-Formatted Regression and Summary St
atistics Tables.
 R package version 5.2.2. https://CRAN.R-project.org/package=stargazer


Attaching package: 'lubridate'

The following object is masked from 'package:hms':

    hms

The following objects are masked from 'package:base':

    date, intersect, setdiff, union

*** This is beta software. Please report any bugs!
*** See the NEWS file for recent changes.

Attaching package: 'reshape2'

The following object is masked from 'package:tidyr':

    smiths
```

We load a dataset which contains simulated data based on a small portion of the PACE-UP trial epoch-level dataset. There are 52 patients in this dataset, each providing seven days' worth of data from their accelerometer. Each row of this dataset represents a 5-second epoch where vector magnitude and step count is measured by the accelerometer. This dataset contains the following variables:

- Patient ID number
- Date
- Day of week
- Day order
- Time at the start of the epoch
- Step count for the duration of the epoch
- Vector magnitude (VM) for the duration of the epoch
- Wear time per day
- Gender
- Age
- BMI at baseline

The values for patient ID number, Gender, Age and BMI at baseline are fabricated, so these data are not identifiable. These data are extracted from GT3X+ accelerometer using the Actilife Software. Typically, a large number of other variables can be extracted such as time spent in different types of activities.

In [16]:

```
Example <- readRDS("Exampledat.RDS")
head(Example)
```

Age	Gender	Date	Day.of.Week	Day_order	Steps.Counts	Weartime_R	Time	Steps	VM
55	F	2014-01-23	Thursday	1	5950	914.5833	00:01:00	0	0
55	F	2014-01-23	Thursday	1	5950	914.5833	00:01:05	0	0
55	F	2014-01-23	Thursday	1	5950	914.5833	00:01:10	0	0
55	F	2014-01-23	Thursday	1	5950	914.5833	00:01:15	0	0
55	F	2014-01-23	Thursday	1	5950	914.5833	00:01:20	0	0
55	F	2014-01-23	Thursday	1	5950	914.5833	00:01:25	0	0

Analysing epoch-level data

Classifying Runs

The calc_runs() classifies epoch-level data into the following possible activity categories: active, sleep, inactive, nonwear and sleep-extra, and identifies the start and end times of each activity. The output provides:

- start: the epoch number when the activity starts
- end: the epoch number when the activity ends
- length: the length, in minutes, of the activity
- type: the type of activity, which can be active, sleep, inactive, nonwear, sleep-extra
- start_date: the date of the start of activity
- start_time: the time of the start of activity
- end_date: the date of the end of the activity
- end_time: the time of the end of the activity

We demonstrate for patient 52 the activites across their 7 days.

In [12]:

```
calc_runs(Example %>% filter(ID=="52"))
```

1.

start	end	length	type	start_date	start_time	end_date	end_time
1	4796	399.66667	sleep	2014-01-23	00:01:00	2014-01-23	06:40:35
4797	15759	913.66667	active	2014-01-23	06:40:40	2014-01-23	21:54:10
15760	21984	518.75000	sleep	2014-01-23	21:54:15	2014-01-24	06:32:55
21985	32047	838.66667	active	2014-01-24	06:33:00	2014-01-24	20:31:30
32048	39603	629.66667	sleep	2014-01-24	20:31:35	2014-01-25	07:01:10
39604	52579	1081.41667	active	2014-01-25	07:01:15	2014-01-26	01:02:30
52580	58257	473.16667	sleep	2014-01-26	01:02:35	2014-01-26	08:55:40
58258	59462	100.50000	active	2014-01-26	08:55:45	2014-01-26	10:36:05
59463	60581	93.25000	nonwear	2014-01-26	10:36:10	2014-01-26	12:09:20
60582	67329	562.41667	active	2014-01-26	12:09:25	2014-01-26	21:31:40
67330	73807	539.83333	sleep	2014-01-26	21:31:45	2014-01-27	06:31:30
73808	79056	437.50000	active	2014-01-27	06:31:35	2014-01-27	13:48:55
79057	80222	97.16667	nonwear	2014-01-27	13:49:00	2014-01-27	15:26:05
80223	85048	402.25000	active	2014-01-27	15:26:10	2014-01-27	22:08:15
85049	91000	496.00000	sleep	2014-01-27	22:08:20	2014-01-28	06:24:15
91001	102766	980.58333	active	2014-01-28	06:24:20	2014-01-28	22:44:45
102767	108369	466.91667	sleep	2014-01-28	22:44:50	2014-01-29	06:31:40
108370	113678	442.50000	active	2014-01-29	06:31:45	2014-01-29	13:54:05
113679	115258	131.66667	nonwear	2014-01-29	13:54:10	2014-01-29	16:05:45
115259	119209	329.33333	active	2014-01-29	16:05:50	2014-01-29	21:35:00
119210	120948	144.91667	sleep	2014-01-29	21:35:05	2014-01-29	23:59:55

Calculating Weartime

The calc_weartime() function calculates the weartime for a specific day. The VM of the day is needed as input. For example, for Patient 52, on their first day using the accelerometer (a Thursday), their weartime is calculated as:

In [13]:

```
P52_Thurs <- Example %>% filter(ID==52 & Day.of.Week=="Thursday")
calc_weartime(P52_Thurs$VM)
```

914.583333333333

We compute weartime for each patient, for each day of wear, and add it as an additional variable in the dataset.

In [19]:

```
Weartime <- Example %>% group_by(ID, Date) %>% summarise(Weartime_R=calc_weartime(VM))
head(Weartime)
Example <- left_join(Example, Weartime, by=c("ID", "Date"))
```

ID	Date	Weartime_R
1	2014-06-07	722.1667
1	2014-06-08	891.8333
1	2014-06-09	756.4167
1	2014-06-10	763.3333
1	2014-06-11	803.5000
1	2014-06-12	775.0833

Visualizing Epoch-level Data

The plot_week() function takes Epoch-level data for a particular patient and plots the Vector Magnitude across each day, and indicates the different activities throughout the day.

In [17]:

```
options(repr.plot.width=5, repr.plot.height=6)
Person1 <- Example %>% filter(ID=="34")
Person2 <- Example %>% filter(ID=="52")
p1 <- plot_week(Person1)
p1
```

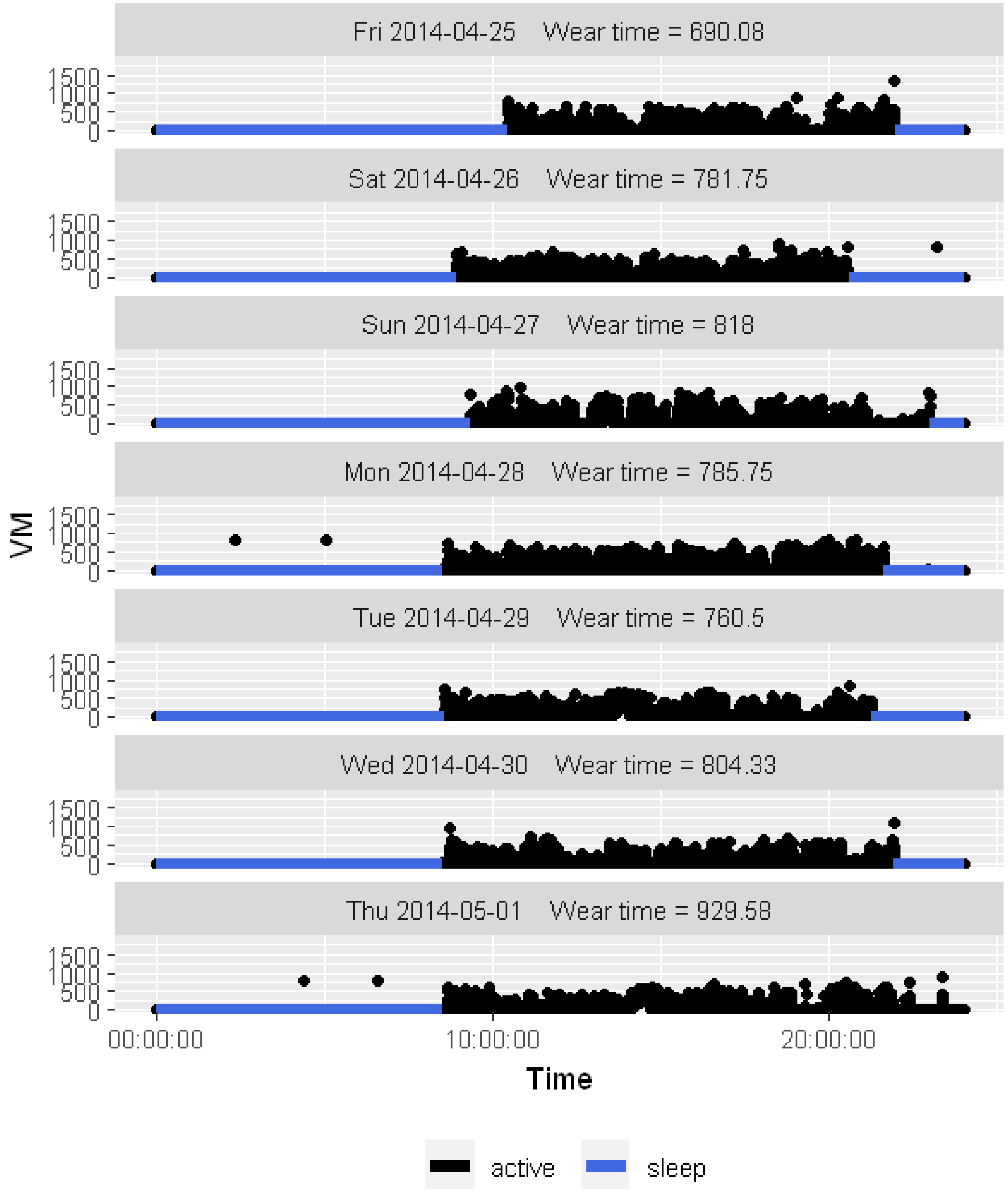

In [18]:

```
p2 <- plot_week(Person2)
p2
```

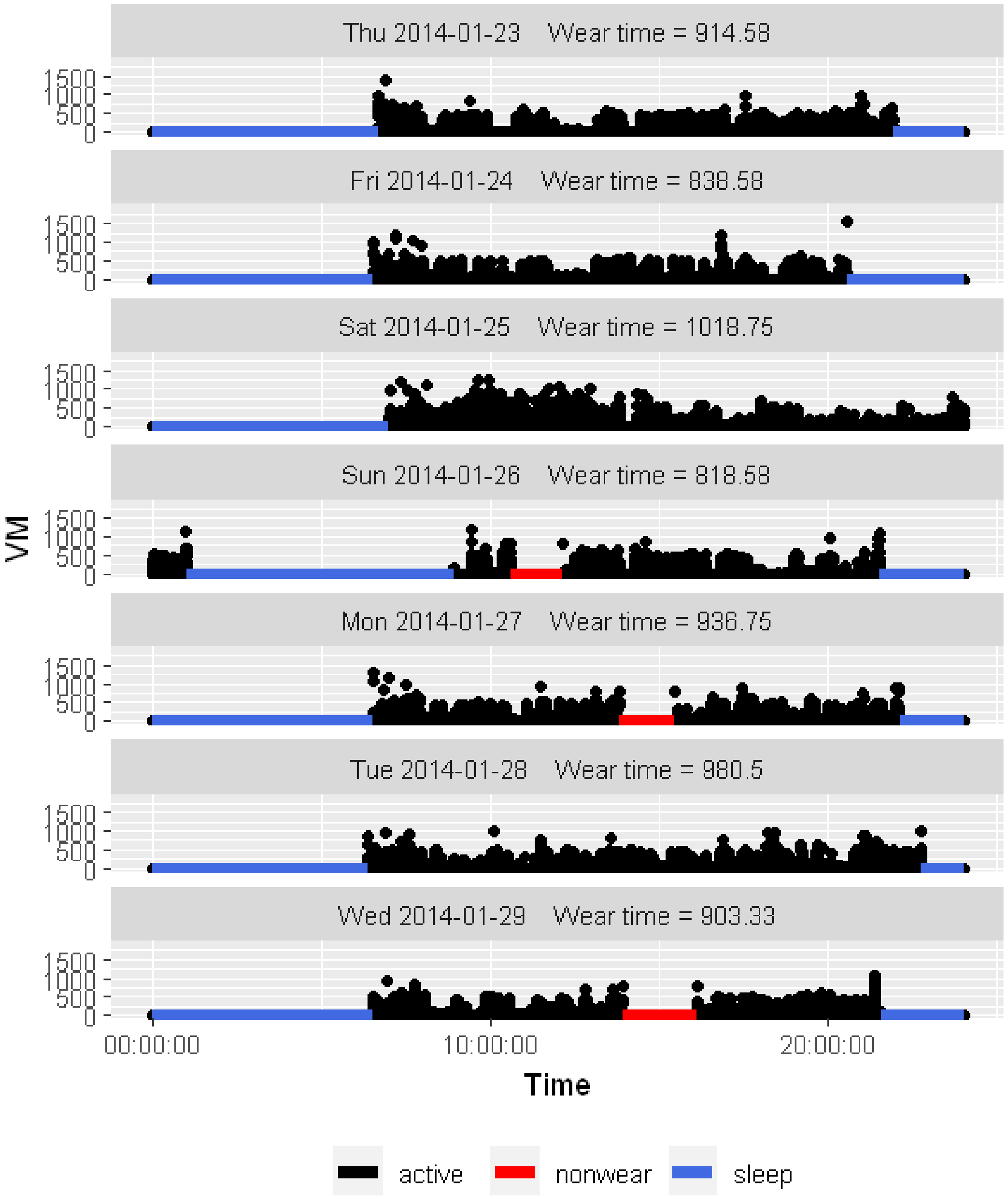

Non-parametric imputation

The impute_and_analyse() function carries out non-parametric imputation. In summary, this function carries out the following tasks:

- Identifies missing periods in the dataset, including nonwear, sleep-extra and whole weeks which need to be imputed.
- Peforms multiple imputation either using self or non-self donation. By default, if there are less than four donors in the donor pool, non-self donation is carried out.
- Exports the following files:

> 1. a file with M copies of the dataset with the total step counts per week for each patient ID. Where there is missingness during the week the M values are different. If there is no missingness, the M values are the same.
> 2. a file with generic and specific upper bounds for daily step counts for each patient ID, which could be used in STATA to do Tobit regression.
> 3. a file with missingness patterns saved for each patient ID, which can be used for further analyses.

- Analyses the data by calculating the mean and standard error of the week-average step counts when non-parametric imputation is used, and when missingness is ignored (available case analysis).

Inputs for this function is as follows:

- **Simdata**: dataset containing epoch-level data.
- **sim**: integer for simulation number. Set to 1 by default.
- **M**: integer greater than 1 for the number of imputations.
- **passive**: function used to transform daily step counts due to skewness. Default is log transformation.
- **path**: path to save upperbounds, which can be used to do parametric imputation in STATA.
- **imputations_save_path**: path to save all imputations for all M. This can be used to analyse data using more complex models.
- **missing_save_path**: path to save missingness classification.
- **missing_save_path**: path to save results for complete case analysis if complete case analysis is performed.
- **adj_base**: set to NULL if average baseline step count is NOT used as a matching variable (default). set to TRUE if used as a matching variables.
- **output**: by default set to TRUE if all output is to be shown. Otherwise set to FALSE.

In [21]:

```
result <- impute_and_analyse(Simdata=Example, sim=1, M=10, passive=log,
                  path=NULL, imputations_save_path=NULL, missing_save_path=NULL,  cc_s
ave_path=NULL,
                             adj_base=NULL, output=FALSE)
result
```

sim	Method	mean	se
1	Ignore	6697.374	396.4339
1	Complete Case	NA	NA
1	Non-para	6978.915	372.0837

In [ ]:
